# Genome-Wide Association Studies for Pasmo Resistance in Flax (*Linum usitatissimum L*.)

**DOI:** 10.3389/fpls.2018.01982

**Published:** 2019-01-14

**Authors:** Liqiang He, Jin Xiao, Khalid Y. Rashid, Zhen Yao, Pingchuan Li, Gaofeng Jia, Xiue Wang, Sylvie Cloutier, Frank M. You

**Affiliations:** ^1^Ottawa Research and Development Centre, Agriculture and Agri-Food Canada, Ottawa, ON, Canada; ^2^Key Laboratory of Crop Genetics and Germplasm Enhancement, College of Agriculture, Nanjing Agricultural University/JCIC-MCP, Nanjing, China; ^3^Morden Research and Development Centre, Agriculture and Agri-Food Canada, Morden, MB, Canada; ^4^Crop Development Centre, University of Saskatchewan, Saskatoon, SK, Canada

**Keywords:** pasmo resistance, quantitative trait loci (QTL), quantitative trait nucleotides (QTNs), fiber, linseed, core collection, flax, *Linum usitatissimum*

## Abstract

Pasmo is one of the most widespread diseases threatening flax production. To identify genetic regions associated with pasmo resistance (PR), a genome-wide association study was performed on 370 accessions from the flax core collection. Evaluation of pasmo severity was performed in the field from 2012 to 2016 in Morden, MB, Canada. Genotyping-by-sequencing has identified 258,873 single nucleotide polymorphisms (SNPs) distributed on all 15 flax chromosomes. Marker-trait associations were identified using ten different statistical models. A total of 692 unique quantitative trait nucleotides (QTNs) associated with 500 putative quantitative trait loci (QTL) were detected from six phenotypic PR datasets (five individual years and average across years). Different QTNs were identified with various statistical models and from individual PR datasets, indicative of the complementation between analytical methods and/or genotype × environment interactions of the QTL effects. The single-locus models tended to identify large-effect QTNs while the multi-loci models were able to detect QTNs with smaller effects. Among the putative QTL, 67 had large effects (3–23%), were stable across all datasets and explained 32–64% of the total variation for PR in the various datasets. Forty-five of these QTL spanned 85 resistance gene analogs including a large toll interleukin receptor, nucleotide-binding site, leucine-rich repeat (TNL) type gene cluster on chromosome 8. The number of QTL with positive-effect or favorite alleles (NPQTL) in accessions was significantly correlated with PR (*R*^2^ = 0.55), suggesting that these QTL effects are mainly additive. NPQTL was also significantly associated with morphotype (*R*^2^ = 0.52) and major QTL with positive effect alleles were present in the fiber type accessions. The 67 large effect QTL are suited for marker-assisted selection and the 500 QTL for effective genomic prediction in PR molecular breeding.

## Introduction

Flax (*Linum usitatissimum* L.) is an important economic crop for both linseed and stem fiber. As of 2011, flax was the third largest textile fiber crop and the fifth largest oil crop in the world, with Canada being the world's largest exporter of flax seeds (You et al., 2017). Pasmo, caused by *Septoria linicola* (Speg.) Garassini, is one of the most widespread diseases threatening flax production. Infected plants show brown circular lesions on leaves and brown to black banding patterns alternating with green healthy tissue on stems. Pasmo infects flax plants from seedling to maturity, but it is most acute during ripening under high humidity and high temperature conditions. During flowering, yield losses in susceptible varieties can reach up to 75% despite fungicide application (Hall et al., 2016). Pasmo also negatively affects seed and fiber quality. Despite the slow improvements made in pasmo resistance (PR) through breeding, developing resistant varieties remains the most efficient and environmentally friendly approach to prevent yield losses caused by the disease.

Conventional breeding approaches have been widely used to incorporate genetic variations to improve agronomic traits and introduce durable resistance to diseases in flax (Soto-Cerda et al., 2014b). The availability of the latest molecular tools allows the rapid identification of genes of interest and the selection of individuals carrying favorable genes, and may well-serve to improve breeding efficiency. Development of molecular markers associated with host resistance to pathogens is paramount to marker-assisted selection (MAS), enhancing the power of selection in plant breeding by combining the advantages of high precision and reduced cost (Kumar et al., 2011). MAS for disease resistance is routinely applied for a number of plant-pathogen systems to select resistant genotypes (Miedaner and Korzun, 2012). To date, no genetic study on flax PR has been reported despite the identification of more than one million single nucleotide polymorphisms (SNPs) from a flax core collection (You et al., unpublished data) that constitute a suitable genotypic dataset to detect marker-trait associations (MTAs) through genome-wide association studies (GWAS).

GWAS commonly estimate the statistical significance of MTAs in a diverse genetic panel that can lead to the identification of causal genes underlying phenotypes. GWAS with high-throughput genotyping are advantageous over traditional biparental population analyses, such as rapid processing of large mapping populations, high abundance of molecular markers, and identification of causal loci at a higher resolution (Goutam et al., 2015; Ogura and Busch, 2015). GWAS have been successfully applied to the identification of MTAs for many important flax agronomic traits (Soto-Cerda et al., 2014a,b; Xie et al., 2017; You et al., 2018b). The effectiveness of GWAS in identifying MTAs for disease resistance traits is exemplified in wheat for fungal diseases, such as *Fusarium* head blight (FHB) (Buerstmayr et al., 2009), leaf and stem rusts (Liu et al., 2017).

In general, population structure can be represented by proportions of individuals from subpopulations, regularly called the Q matrix (Larsson et al., 2013), or alternatively principal components (PCs) (Reich et al., 2008; Stich et al., 2008; Zhang et al., 2009) derived from genome-wide molecular markers. The relationships among individuals of a population are represented by a kinship matrix (K). False positive MTAs generally result from two indirect factors: population structure and kinship among individuals (Price et al., 2006; Liu et al., 2016). Two statistical models have been widely used to reduce false positives. The first is the General Linear Model (GLM) or Q model (Price et al., 2006) in which the population structure is fitted as fixed effect. The second is the Mixed Linear Model (MLM) (Yu et al., 2006) that additionally fits kinship as random effect, hence its alternative name, the Q + K model. Theoretically, MLM methods correct the inflation from small polygenic effects, effectively controlling the population stratification bias (Wen et al., 2017); thus, some reports show that the Q + K model outperforms the independent Q and K only models (Liu et al., 2016). The computational burden of MLMs remains a major issue. Some methods have been proposed to improve computational efficiency including Efficient Mixed-Model Association (EMMA) (Kang et al., 2008) and Genome-Wide Efficient Mixed-Model Association (GEMMA) (Zhou and Stephens, 2012).

GLM and MLMs are single-locus methods that perform one-dimensional genome scans by testing one marker at a time using stringent multiple test corrections (such as Bonferroni) as significance threshold. As such, these methods have relatively low power to detect the polygenes with small effects that underlay most quantitative traits. Thus, Multi-Locus Mixed-Model (MLMM) (Segura et al., 2012) was proposed to simultaneously test multiple markers. Alternative and powerful multi-locus methods have been proposed to identify quantitative trait nucleotides (QTNs) with small effects, such as the multi-locus random-SNP-effect Mixed Linear Model (mrMLM) (Wang et al., 2016; Li et al., 2017), the FAST multi-locus random-SNP-effect EMMA (FASTmrEMMA) (Wen et al., 2017), the polygene-background-control-based Least Angle Regression plus Empirical Bayes (pLARmEB) (Zhang et al., 2017), the Iterative modified-Sure Independence Screening EM-Bayesian LASSO (ISIS EM-BLASSO) (Tamba et al., 2017), and the integration of Kruskal–Wallis test with Empirical Bayes under polygenic background control (pKWmEB). These multi-locus methods do not rely on stringent Bonferroni correction (Ren et al., 2017); the algorithms underlying these statistical models substantially increase the statistical power and reduce Type 1 error and running time (Wang et al., 2016; Li et al., 2017; Ren et al., 2017; Tamba et al., 2017; Wen et al., 2017; Zhang et al., 2017). An additional multi-locus model, called Fixed and random model Circulating Probability Unification (FarmCPU) (Liu et al., 2016) divides the MLMM into a fixed effect model (FEM) and a random effects model (REM) and uses them iteratively. Its advantages are improved statistical power and reduction of the confounding between population structure, kinship, and QTN (Liu et al., 2016).

To find QTL associated with field PR, we performed GWAS using a diverse genetic panel of 370 accessions of the flax core collection (Diederichsen et al., 2012; Soto-Cerda et al., 2013) and 258,873 SNPs identified from this population (You et al., unpublished data). Seven multi-locus and three single-locus statistical methods were evaluated with the PR datasets from 5 consecutive years to determine the suitable statistical methods for detecting putative QTL with large or small effects and environmental stability.

## Materials and Methods

### Genetic Panel for GWAS

A diverse genetic panel of 370 cultivated flax accessions from the core collection (Diederichsen et al., 2012; Soto-Cerda et al., 2013) was used. The core collection was assembled from the world collection of 3,378 flax accessions, collected from 39 countries and corresponding to 11 geographical origins defined as North America, South America, Eastern Asia, Western Asia, Southern Asia, Central, and Eastern Europe, Western Europe, Southern Europe, Northern Europe, Oceania, and Africa. This panel contained 17 landraces, 85 breeding lines, 232 cultivars, and 36 accessions of unknown improvement status that were grouped into two morphotypes: 80 fiber and 290 linseed (You et al., 2017).

### Phenotyping of Pasmo Resistance and Statistical Analysis

The 391 accessions were evaluated for field PR in the same pasmo nursery from 2012 to 2016 at Agriculture and Agri-Food Canada, Morden Research and Development Center's farm, Morden, Manitoba, Canada. A type-2 modified augmented design (MAD2) (Lin and Poushinsky, 1985) was used for the field trials (You et al., 2017). Each accession was seeded during the second or third week of May every year. Approximately 200 g of pasmo-infested chopped straw from the previous growing season was spread between rows as inoculum when plants were ~30-cm tall. A misting system was operated for 5 min every half hour for 4 weeks, except on rainy days, to ensure conidia dispersal and disease infection and development. PR was assessed on leaves and stems of all plants in a single row plot using a pasmo severity (PS) scale of 0–9 (Table 1). Field assessments were conducted at the early (P1) and late flowering stages (P2, 7–10 days after P1), the green boll stage (P3, 7–10 days after P2), and the early brown boll stage (P4, 7–10 days after P3). In 2014 and 2015, only the first three field assessments were conducted because early maturity of the plants did not allow for a fourth rating. A rating of 0–2 is considered resistant (R), 3–4 moderately resistant (MR), 5–6 moderately susceptible (MS), and 7–9 susceptible (S). The statistical analysis for the phenotypic data was performed as described in You et al. (2013). A total of 370 accessions that had complete phenotypic data and sequence data were used for GWAS (Table S1).

**Table 1:** Criteria for field assessment of pasmo severity on a scale of 0–9.

**Severity score**	**Criteria**
0	No sign of pasmo, the most vigorous
1	< 10 leaf area or/and stem area affected by pasmo
2	10–20% leaf area or/and stem area affected by pasmo
3	21–30% leaf area or/and stem area affected by pasmo
4	31–40% leaf area or/and stem area affected by pasmo
5	41–50% leaf area or/and stem area affected by pasmo
6	51–60% leaf area or/and stem area affected by pasmo
7	61–70% leaf area or/and stem area affected by pasmo
8	71–80% leaf area or/and stem area affected by pasmo
9	>80% leaf area or/and stem area affected by pasmo

*Assessment of pasmo severity is based on all plants in a single row plot*.

The variance components for pasmo severity were estimated using the linear mixed effects “lmer” model in R package “lme4.” All effects of variance components were treated as random and the following linear model was used:

$$\begin{array}{lll} X_{ij}=\mu +G_{i}+Y_{j}+{\left(GY\right)}_{ij}+\varepsilon_{ij},\;i\; & = & \;1,2,\ldots ,n\;and\\ \;j\; & = & \;1,2,\ldots ,m, \end{array}$$

where *n* and *m* are the number of genotypes and years, respectively, *X*_*ij*_ is the observed pasmo severity, μ is the overall mean, *G*_*i*_ is the effect resulting from the *i*th genotype, *Y*_*j*_ is the effect resulting from the *j*th year, *(GY)*_*ij*_ is the effect resulting from genotype × year (environment) interaction, and ε_*ij*_ is the residual error (effect resulting from the experimental error).

### Resequencing and SNP Discovery of the Core Collection

Genotyping by sequencing (GBS) methodology was employed to genotype all individuals of the core collection. The Illumina HiSeq 2000 platform (Illumina Inc., San Diego, USA) was used to generate 100-bp paired-end reads with ~15.5 × genome equivalents of the reference genome. All reads from each individual of the population were aligned to the scaffold sequences of the flax reference genome (Wang et al., 2012) using BWA v0.6.1(Jo and Koh, 2015) with base-quality Q score in Phred scale >20 and other default parameters. The alignment file for each individual was used as input for SNP discovery using the software package SAMtools (Li et al., 2009). All variants were further filtered to get a set of high-quality SNPs as previously described (Kumar et al., 2012). The coordinates of SNPs were then converted to the chromosome scale of the flax pseudomolecules v2.0 upon its release (You et al., 2018a). All procedures were implemented in the AGSNP pipeline (You et al., 2011, 2012) and its updated GBS version (Kumar et al., 2012). The detected SNPs were further filtered with minor allele frequency (MAF) > 0.05 and SNP genotyping rate ≥ 60%. To minimize contribution from regions of extensive strong linkage disequilibrium (LD), a single SNP was retained per 200-kb window when pairwise correlation coefficients (*r*^2^) among neighboring SNPs were >0.8 (International HapMap, 2005; Huang et al., 2010), resulting in a total of 258,873 SNPs. Missing SNPs (on average 14.13% of a missing data rate) were imputed using Beagle v.4.2 with default parameters (Browning and Browning, 2007).

### Genome-Wide Association Study and Validation

GWAS analyses were conducted separately for combinations of the 5 individual years and the 5-years average datasets with 10 single- and multi-locus methods (Table 2). Kinship genetic relationship matrices were estimated using the protocol suggested by each GWAS software package. The population structure of the 370 accessions was estimated using Frappe (http://med.stanford.edu/tanglab/software/frappe.html) or PCs as determined by principal component analysis (PCA) using MVP in the R package (https://github.com/XiaoleiLiuBio/MVP). Using Frappe, the 370 accessions of the flax core collection were grouped into five sub-populations that corresponded to two major morphotypes (fiber and oil) and different geographical regions (Table S1).

**Table 2:** Statistical methods used for GWAS.

**Statistical model**	**Q matrix or PCs**	**Threshold for QTNs**	**GWAS software**	**References**
GLM	First six PCs	Bonferroni correction	MVP v1.0.1	Price et al., 2006
MLM	First six PCs	Bonferroni correction	MVP v1.0.1	Yu et al., 2006
FarmCPU	First six PCs	Bonferroni correction	MVP v1.0.1	Liu et al., 2016
GEMMA	None needed	Bonferroni correction	GEMMA v0.96	Zhou and Stephens, 2012
mrMLM	From Frappe	LOD ≥ 3	mrMLM v3.0	Wang et al., 2016
FASTmrEMMA	From Frappe	LOD ≥ 3	mrMLM v3.0	Wen et al., 2017
ISIS EM-BLASSO	From Frappe	LOD ≥ 3	mrMLM v3.0	Tamba et al., 2017
pLARmEB	From Frappe	LOD ≥ 3	mrMLM v3.0	Zhang et al., 2017
pKWmEB	From Frappe	LOD ≥ 3	mrMLM v3.0	Ren et al., 2017
FASTmrMLM	From Frappe	LOD ≥ 3	mrMLM v3.0	https://cran.r-project.org/web/packages/mrMLM/index.html

*The kinship matrix used for each method was calculated using the module implemented in the corresponding software. PC, principal component; QTN, quantitative trait nucloetide*.

For GLM, MLM and FarmCPU, the first six PCs, accounting for 33.04% of the total variation, were chosen as covariates to measure population structure (Figure S1). GEMMA was also compared with the regular MLM methods because it does not require a Q matrix. The threshold of significant associations for all four of these methods was determined by a critical *P*-value (α = 0.05) subjected to Bonferroni correction, i.e., the corrected *P*-value = 1.93 × 10^−7^ (0.05/258,873 SNPs). GWAS analyses for the GLM, MLM, and FarmCPU were performed using the R package MVP (https://github.com/XiaoleiLiuBio/MVP) and for GEMMA using the GEMMA software (https://github.com/genetics-statistics/GEMMA). The additional six multi-locus methods were conducted with default parameters using the R package mrMLM (https://cran.r-project.org/web/packages/mrMLM/index.html) (Table 2). Because these six methods are implemented in the same mrMLM R package and developed by the same research team, we refer to them as “mrMLM methods.” A log of odds (LOD) score of three was used to detect robust association signals for these six methods.

**Figure 1:**
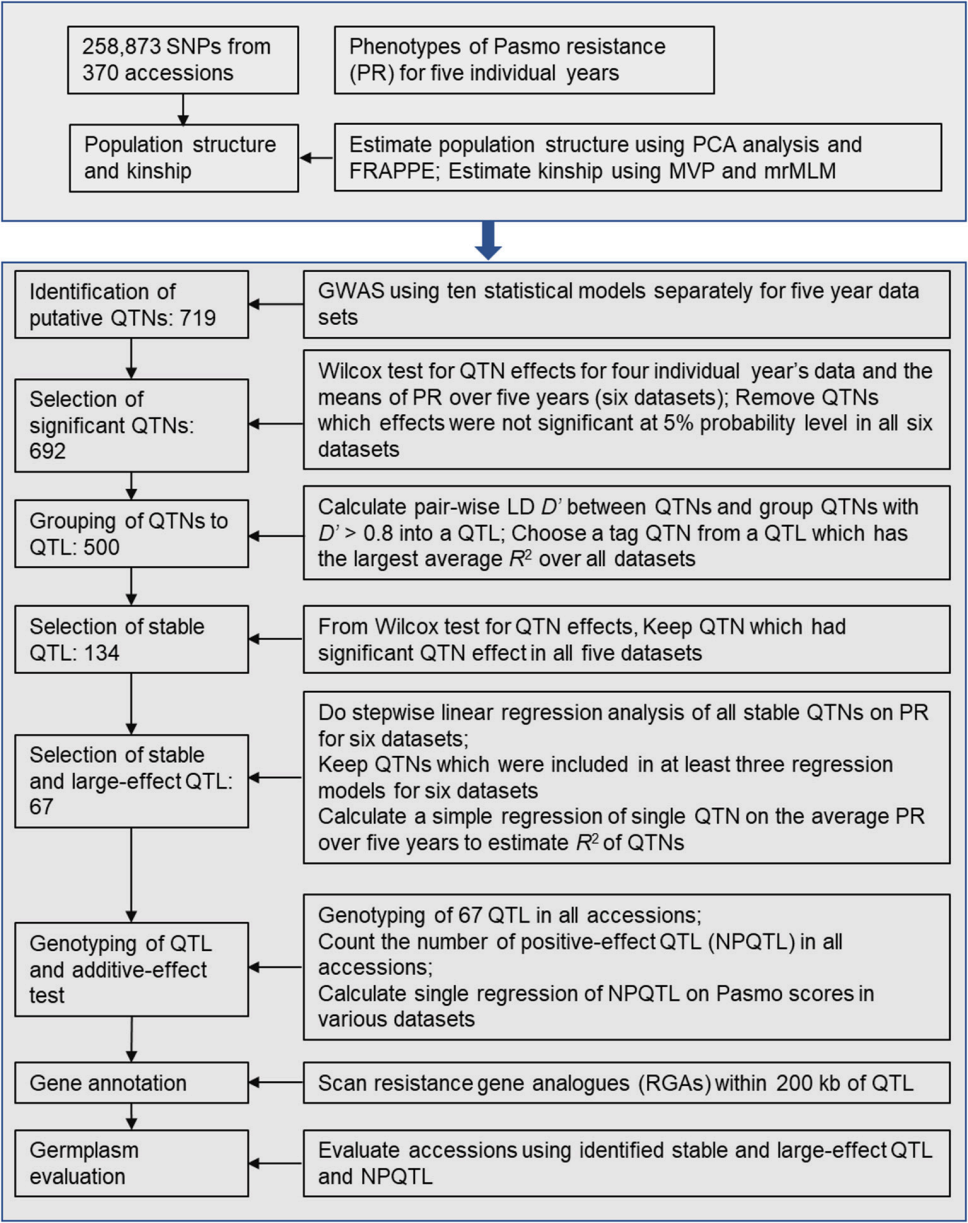
Diagram of stable and large-effect quantitative trait loci (QTL) associated with flax pasmo resistance. NPQTL: the number of QTL with positive-effect alleles.

After putative QTNs were identified, we performed QTN analysis to obtain sets of QTNs/QTL. The procedure is summarized in Figure 1. First, we tested the significance of the difference in PS between two alleles of a QTN (henceforth called QTN effect) in all accessions. Statistically significant differences served to validate the QTNs. Wilcox non-parametric tests were performed using the R function *wilcox.test* to remove the non-significant QTNs at a 5% probability level. The direction (positive or negative) of QTN effects were subsequently assessed. Only QTNs with consistent effect directions in all PS datasets were considered valid and were retained. Such QTNs were grouped into QTL by calculating linkage disequilibrium (D′) between pairs of QTNs on the same chromosomes using plink v1.9 (https://www.cog-genomics.org/plink2). Neighboring QTNs with *D*′ > 0.8 were grouped into the same QTL (Twells et al., 2003; Grassmann et al., 2017). For each such defined QTL, the QTN of the largest average *R*^2^ over all datasets was chosen as a representative or tag for the QTL. *R*^2^ were calculated based on simple regressions of QTNs on PS because they represent the proportion of the total variation of PS explained by the QTNs/QTL.

Statistically stable QTL were those significant across all six PS datasets. Multiple regressions of all stable QTL were fitted to each of the six PS datasets using a forward stepwise regression to select QTL with significant contributions to PS. Six regression models were obtained for the six PS datasets. Only QTL existing in at least three regression models were considered to be statistically stable with relatively large effects.

To test QTL effect additivity, the number of QTL with positive-effect or favorite alleles (NPQTL) in all accessions was tallied. A QTL with positive-effect or favorite allele (PQTL) in a given accession was called if this accession possessed a positive effect allele for that QTL. In the case of the PS trait (PS rating is opposite to resistance), alleles with positive signs are associated with lower PR. A simple regression of NPQTL on PS in the population was calculated. Correlations of NPQTL with PS in the six PS datasets were calculated using the R function “*cor*.”

### Resistance Gene Analogs (RGAs) Co-localized With QTL

A total of 1,327 RGAs have been identified in the flax pseudomolecule (You et al., 2018a). To predict candidate resistance genes co-localized with QTL, the RGAs within 200 kb of a QTL's flanking region were considered.

### Evaluation of the Flax Core Collection

The extreme pasmo resistant and susceptible accession subsets and all 370 accessions were evaluated based on the identified stable and large-effect QTL. Two extreme subsets of 23 resistant (R) and 23 susceptible (S) were selected based on PS ratings. Two-dimensional cluster analysis for accessions and the QTL were performed. The Euclidean distances between accessions or between QTL were calculated based on QTL genotypes (positive alleles as 1 and alternate alleles as 2) using the “*dist*” function with the “*euclidean*” method in R. The Ward algorithm in the function “*hclust*” of the R package stats was employed for hierarchical cluster analysis. Dendrograms and heat maps were created with the R package Complexheatmap.

## Results

### Pasmo Resistance

**Figure 2:**
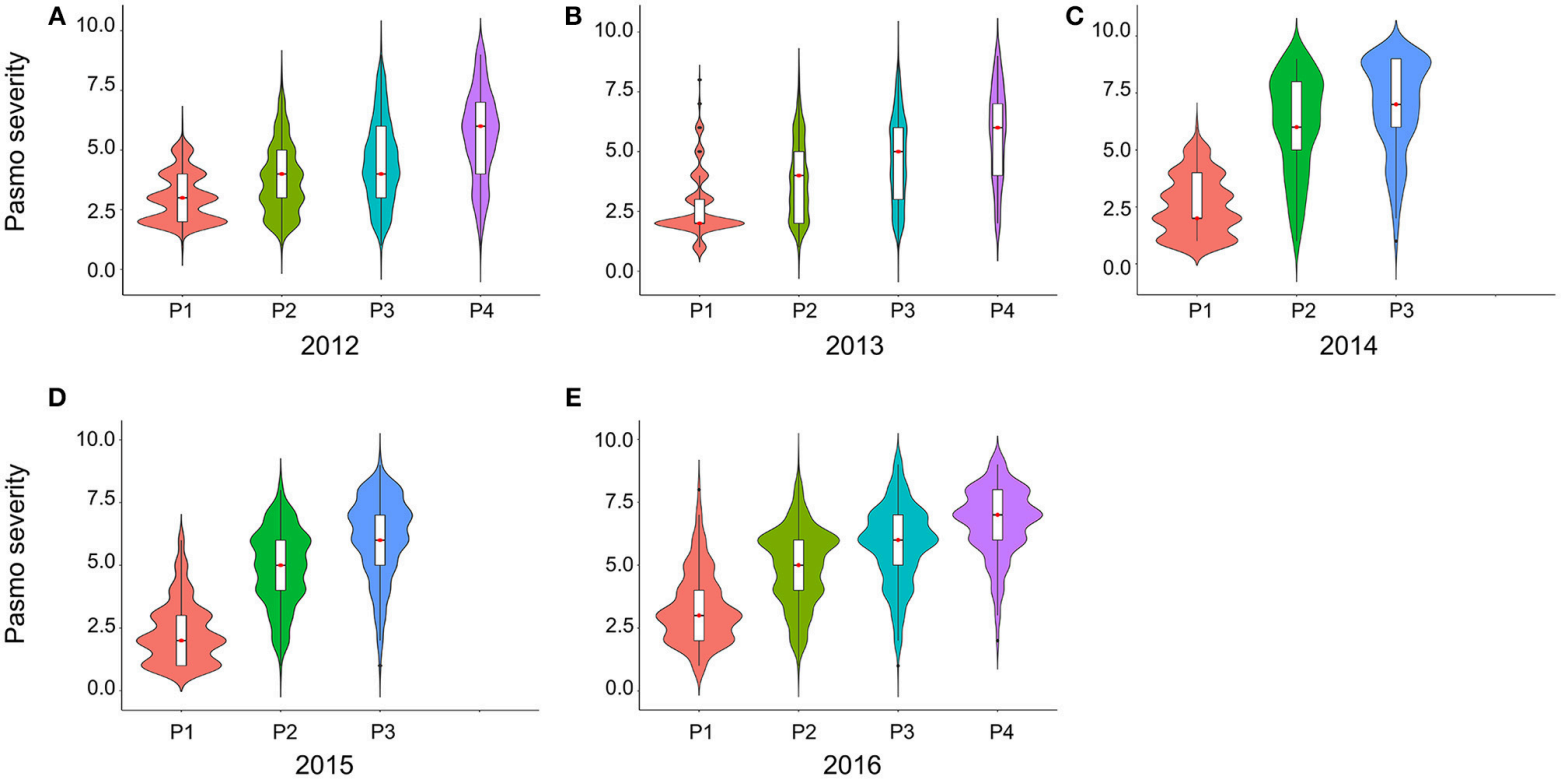
Violin plots of pasmo severity observed in various growth stages in 2012 **(A)**, 2013 **(B)**, 2014 **(C)**, 2015 **(D)**, and 2016 **(E)**.

PS ratings increased with growth stage and peaked at the final evaluation stage every year (Figure 2; Table S2), supporting the adoption of the final stage data for analysis. Significant correlations of PS ratings across years were observed (Table 3) but were largely affected by year and genotype × year interaction (Table S3). The variance of genotype × year interaction accounted for 24.23% of the total variation of PS (Table S4). Thus, datasets from all individual years and the 5-years average were used for GWAS analyses.

**Table 3:** Spearman correlation coefficients of pasmo severity between years (2012–2016).

	**2012**	**2013**	**2014**	**2015**	**2016**
2012	1	0.56^**^	0.54^**^	0.44^**^	0.62^**^
2013		1	0.51^**^	0.49^**^	0.60^**^
2014			1	0.76^**^	0.70^**^
2015				1	0.70^**^
2016					1

** *Significant at 1% probability level*.

### Identification of PR QTL

A total of 719 putative QTNs were identified using the three single-locus and seven multi-locus methods for the six PS datasets (Table S4). To further statistically check the significance of QTNs, a Wilcox non-parametric test was conducted for the six datasets separately. A total of 27 QTNs were removed because they were not significant in all six datasets. The remaining 692 QTNs were merged into a total of 500 QTL based on the linkage disequilibrium *D*′ criteria between contiguous QTNs. GLM detected multiple significant QTNs in the same QTL region while in most cases a QTN corresponded to a QTL for other single-locus and multi-locus methods (Table 4). Tag QTNs were selected to represent each QTL for downstream analyses.

**Table 4:** Comparison of QTN/QTL identification for different statistical models.

**Statistical**	**No. of**	**No. of**	**No. of non-**	**Average**	***R*^**2**^**
**model**	**QTL**	**QTNs**	**significant**	***R*^**2**^ (%)**	**range (%)**
	**identified**	**identified**	**QTNs**		
GLM	209	346	2	5.57	0.48–15.02
MLM	1	1	0	15.02	N/A
GEMMA	6	6	0	11.13	3.59–15.02
mrMLM	97	99	7	2.75	0.36–15.02
FASTmrEMMA	60	62	2	2.82	0.25–6.92
ISIS EM-BLASSO	97	98	8	2.91	0.29–12.68
pLARmEB	118	120	8	2.69	0.22–12.68
pKWmEB	95	95	5	2.93	0.25–12.68
FASTmrMLM	125	125	3	2.69	0.25–15.02
FarmCPU	22	22	3	5.09	0.42–15.02

Of the three single-locus methods, MLM identified only one QTL (*R*^2^ = 15.02%) while GEMMA detected six with an average *R*^2^ of 11.13%. GLM identified the largest number of QTL (209) or QTNs (346) of all methods and these had relatively large effects with an average *R*^2^ of 5.57%, ranging from 0.48 to 15.02%.

QTL differed depending on the statistical methods. QTL detected by at least two methods accounted for a small proportion of overall QTL (Tables S4, S5). The mrMLM methods detected more common QTL than the other methods, e.g., 45 QTL in common with pLARmEB and FASTmrMLM and 32 with ISIS EM-BLASSO and pKWmEB (Table 5). Multi-locus methods detected more small-effect QTL than the single-locus methods (Table 4). Six mrMLM methods could identify more QTL with smaller effects (average *R*^2^ of 2.80%) than FarmCPU (average *R*^2^ of 5.09%) owing to the high stringency of the Bonferroni correction used in FarmCPU. Generally, QTL with large effects were identified by both GLM and mrMLM methods (Table S4).

**Table 5:** Number of common QTNs and QTL identified by any two statistical models.

**Statistical model**	**GLM**	**MLM**	**GEMMA**	**FASTmrEMMA**	**FASTmrMLM**	**ISIS EM-BLASSO**	**mrMLM**	**pKWmEB**	**pLARmEB**
MLM	1 (1)								
GEMMA	6 (6)	1 (1)							
FASTmrEMMA	8 (5)	0 (0)	0 (0)						
FASTmrMLM	17 (12)	1 (1)	2 (2)	26 (18)					
ISIS EM-BLASSO	18 (15)	0 (0)	3 (3)	13 (9)	33 (23)				
mrMLM	18 (11)	1 (1)	5 (5)	17 (14)	33 (27)	18 (13)			
pKWmEB	24 (17)	0 (0)	3 (3)	17 (12)	34 (30)	34 (32)	27 (19)		
pLARmEB	20 (13)	0 (0)	2 (2)	26 (15)	50 (45)	31 (20)	29 (24)	32 (27)	
FarmCPU	13 (13)	1 (1)	2 (2)	1 (1)	3 (3)	5 (4)	7 (6)	5 (4)	5 (4)

*Values in parentheses are the number of QTL corresponding to QTNs*.

QTL also differed across individual year datasets (Tables 6, 7, S5) but most (240) were identified from the mean dataset which comprised two to four times more QTL than the individual year datasets (Table 6). This is indicative of strong gene × environment interactions and reinforces the representability of the mean dataset for QTL identification.

**Table 6:** QTNs/QTL for pasmo severity identified using phenotypic data from 5 consecutive years and their mean with ten statistical models.

**Dataset**	**No. of**	**No. of**	**No. of non-**	**Average**	***R*^**2**^**
	**QTL**	**QTNs**	**significant**	***R*^**2**^ (%)**	**range (%)**
	**identified**	**identified**	**QTNs**		
Mean	240	362	4	5.26	0.28–15.02
2012	82	98	12	3.68	0.22–15.02
2013	92	100	8	3.26	0.32–15.02
2014	114	138	7	4.7	0.42–15.02
2015	65	72	4	2.86	0.27–15.02
2016	85	100	3	4.46	0.39–15.02

**Table 7:** Number of common QTNs/QTL identified from any two datasets.

**Dataset**	**mean**	**2012**	**2013**	**2014**	**2015**
2012	33 (30)				
2013	28 (25)	6 (4)			
2014	62 (52)	16 (11)	11 (10)		
2015	18 (14)	3 (2)	5 (2)	6 (3)	
2016	45 (36)	10 (6)	7 (6)	14 (11)	7 (7)

*Values in parentheses are the number of QTL corresponding to QTNs*.

### Validation of PR QTL

Additional validation was warranted for the identified QTNs/QTL. Of the 500 QTL, 134 were detected in all six PS datasets and explained 27.4–60.9% of the total variation; they are considered stable. By construction of forward stepwise multiple regression models, 67 out of the 134 stable QTL were detected in at least three regression models; they explained 31.5–64.2% of the total variation in individual datasets: this is comparable or slightly greater than that of the 134 QTL set. The 67 QTL subset represents the non-redundant and large effect QTL as each of them could explain 3.3–23.4% of the total variation (Table 8). QTL with similar contributions but that were highly correlated and/or that had small effects were excluded.

**Table 8:** Stable and large-effect QTL associated with pasmo resistance.

**QTL**	**Tag QTN**	**Chr**	**Pos**	**SNP**	**Effect**	***R^**2**^***	**Gene/annotation**
1	Lu1-9232234	1	9232234	G/A	−0.91	16.17	
2	Lu1-28707496	1	28707496	G/A	−0.54	5.70	*Lus10006052*/RLK, *Lus10006056/*RLK, *Lus10006057*/RLK, *Lus10006067*/RLK
3	Lu2-3803775	2	3803775	C/T	0.36	3.32	
4	Lu3-19643168	3	19643168	G/A	−1.97	12.82	*Lus10008221*/TNL, *Lus10008222*/TNL, *Lus10008230*/RLP
5	Lu3-20781286	3	20781286	A/C	−1.83	14.63	
6	Lu3-22688547	3	22688547	C/G	−0.89	8.98	*Lus10033291*/RLK
7	Lu4-37769	4	37769	G/A	−1.36	11.23	
8	Lu4-13306407	4	13306407	A/G	0.89	4.58	*Lus10015729*/RLK
9	Lu4-13779313	4	13779313	T/C	−1.73	13.72	
10	Lu4-14576826	4	14576826	A/G	0.42	7.99	*Lus10041509*/RLK, *Lus10041512*/TM-CC
11	Lu4-14615685	4	14615685	A/T	−0.65	10.85	*Lus10041509*/RLK, *Lus10041512*/TM-CC
12	Lu4-14738243	4	14738243	G/T	−0.61	12.64	
13	Lu4-17204590	4	17204590	C/A	0.64	5.17	*Lus10004040*/RLK, *Lus10009107*/TNL, *Lus10009108*/TX, *Lus10009109*/NBS, *Lus10020794*/TM-CC
14	Lu4-17214936	4	17214936	G/T	0.70	5.81	*Lus10004040*/RLK, *Lus10009107*/TNL, *Lus10009108*/TX, *Lus10009109*/NBS, *Lus10020779*/CNL, *Lus10020794*/TM-CC
15	Lu5-1554121	5	1554121	T/A	−0.67	7.75	*Lus10004719*/TNL, *Lus10004726*/CNL, *Lus10004727*/TN
16	Lu5-1650980	5	1650980	C/G	−0.81	6.61	*Lus10004719*/TNL, *Lus10008486*/RLK, *Lus10008491*/RLK
17	Lu5-3575865	5	3575865	C/G	−0.49	9.64	
18	Lu5-4604607	5	4604607	A/G	−0.56	6.58	*Lus10034787*/TM-CC, *Lus10034790*/RLK, *Lus10034795*/RLK
19	Lu5-4858045	5	4858045	C/T	−1.87	12.83	
20	Lu5-13500692	5	13500692	G/A	−1.40	11.9	*Lus10029802*/RLK, *Lus10029810*/TX
21	Lu6-2081466	6	2081466	T/C	0.68	8.30	*Lus10017611*/RLK
22	Lu6-5837358	6	5837358	C/T	−1.18	9.36	
23	Lu6-14738507	6	14738507	C/T	−2.01	13.34	*Lus10014441*/RLP
24	Lu6-15455712	6	15455712	A/G	−1.42	9.63	*Lus10021003*/RLK, *Lus10021022*/RLK
25	Lu6-15506450	6	15506450	A/G	−1.81	12.62	*Lus10021022*/RLK
26	Lu7-2452981	7	2452981	C/T	−0.53	6.30	*Lus10012159*/RLK
27	Lu7-2453965	7	2453965	T/C	−0.56	7.03	*Lus10012159*/RLK
28	Lu7-2491132	7	2491132	G/A	−0.56	8.05	*Lus10012159*/RLK
29	Lu8-14317356	8	14317356	A/T	−0.98	14.32	*Lus10016612*/RLP, *Lus10016620*/RLK
30	Lu8-15830073	8	15830073	C/T	−0.82	8.48	
31	Lu8-15837449	8	15837449	A/T	−1.20	8.24	
32	Lu8-15841885	8	15841885	T/C	−1.15	8.35	
33	Lu8-15963249	8	15963249	A/G	−1.70	14.22	
34	Lu8-16366918	8	16366918	C/T	−1.38	10.90	*Lus10022340*/RLK, *Lus10022345*/RLK, *Lus10022351*/CNL
35	Lu8-17270785	8	17270785	C/G	−1.08	9.59	*Lus10000591*/TM-CC
36	Lu8-17749357	8	17749357	G/A	−1.23	10.16	*Lus10011039*/RLP, *Lus10011064*/RLP
37	Lu8-18251174	8	18251174	G/A	−1.45	10.38	*Lus10007812*/TNL, *Lus10007813*/TNL, *Lus10007814*/TNL, *Lus10007821*/TNL, *Lus10007822*/TNL, *Lus10007823*/OTHER, *Lus10007825*/TNL, *Lus10007826*/TNL, *Lus10007828*/TNL, *Lus10007829*/OTHER, *Lus10007830*/NL, *Lus10007831*/TNL, *Lus10007836*/TNL, *Lus10007852*/TX
38	Lu8-18447612	8	18447612	T/C	−1.41	11.66	*Lus10007790*/TNL, *Lus10007795*/TM-CC, *Lus10007808*/TNL, *Lus10007809*/NL, *Lus10007810*/TNL, *Lus10007811*/TNL, *Lus10007812*/TNL, *Lus10007813*/TNL, *Lus10008540*/RLK
39	Lu8-23104696	8	23104696	C/A	−1.80	16.53	*Lus10018470*/TX
40	Lu8-23142500	8	23142500	T/C	−1.56	13.34	*Lus10018459*/RLK, *Lus10018470*/TX
41	Lu9-1258326	9	1258326	T/A	−1.62	16.01	
42	Lu9-1430465	9	1430465	G/C	−0.69	10.76	*Lus10004333*/RLK
43	Lu9-1896658	9	1896658	G/A	−1.94	17.12	
44	Lu9-4333365	9	4333365	C/A	−2.22	23.39	*Lus10040315*/TM-CC
45	Lu9-6270376	9	6270376	A/G	−0.81	14.34	*Lus10031043*/RLK, *Lus10031058*/TM-CC
46	Lu9-15527375	9	15527375	G/A	−1.07	6.76	
47	Lu9-16348319	9	16348319	C/T	−0.37	4.64	
48	Lu9-19857367	9	19857367	G/A	−1.70	12.67	*Lus10011917*/RLK
49	Lu10-8700793	10	8700793	A/G	−0.53	12.10	*Lus10039958*/RLP
50	Lu11-3330783	11	3330783	A/T	−1.11	7.09	*Lus10042097/*TM-CC
51	Lu12-474480	12	474480	C/T	0.51	8.33	*Lus10020016*/CNL
52	Lu12-1621325	12	1621325	T/A	−1.90	9.41	*Lus10023391*/RLK
53	Lu12-2719326	12	2719326	C/T	−0.62	9.90	*Lus10006971*/TM-CC
54	Lu12-5552631	12	5552631	C/A	0.92	7.10	
55	Lu12-5795458	12	5795458	A/G	0.54	9.67	*Lus10037786*/TM-CC
56	Lu12-5819991	12	5819991	C/G	0.35	6.90	*Lus10037786*/TM-CC
57	Lu12-15686833	12	15686833	A/G	−1.65	13.90	
58	Lu12-16056974	12	16056974	A/C	−1.26	11.26	*Lus10043083*/RLK
59	Lu12-16358216	12	16358216	G/A	0.32	4.25	*Lus10027856*/RLP
60	Lu13-1919638	13	1919638	G/A	−1.55	13.67	*Lus10026845*/TX
61	Lu13-2016767	13	2016767	C/G	−0.38	5.12	*Lus10026845*/TX
62	Lu13-11860250	13	11860250	G/A	−0.96	9.65	
63	Lu13-13051094	13	13051094	G/C	0.68	7.96	
64	Lu13-14299019	13	14299019	A/G	0.39	8.28	*Lus10034637*/RLK, *Lus10034642*/RLK
65	Lu15-976617	15	976617	T/A	−1.65	16.08	*Lus10011216*/TX, *Lus10011223*/RLK, *Lus10011229*/TM-CC
66	Lu15-995626	15	995626	T/A	−0.44	6.27	*Lus10011216*/TX, *Lus10011223*/RLK, *Lus10011229*/TM-CC
67	Lu15-8714776	15	8714776	C/G	−0.98	15.04	

*RLK, receptor-like protein kinase; RLP, receptor-like protein; TM-CC, transmembrane coiled-coil protein; NBSl, nucleotide-binding site domain; LRR, leucine-rich repeat; Toll/interleukin-1 receptor-like domain; CNL, CC–NBS–LRR; TNL, TIR-NBS-LRRs; TN, TIR–NBS; TX, TIR–unknown*.

**Figure 3:**
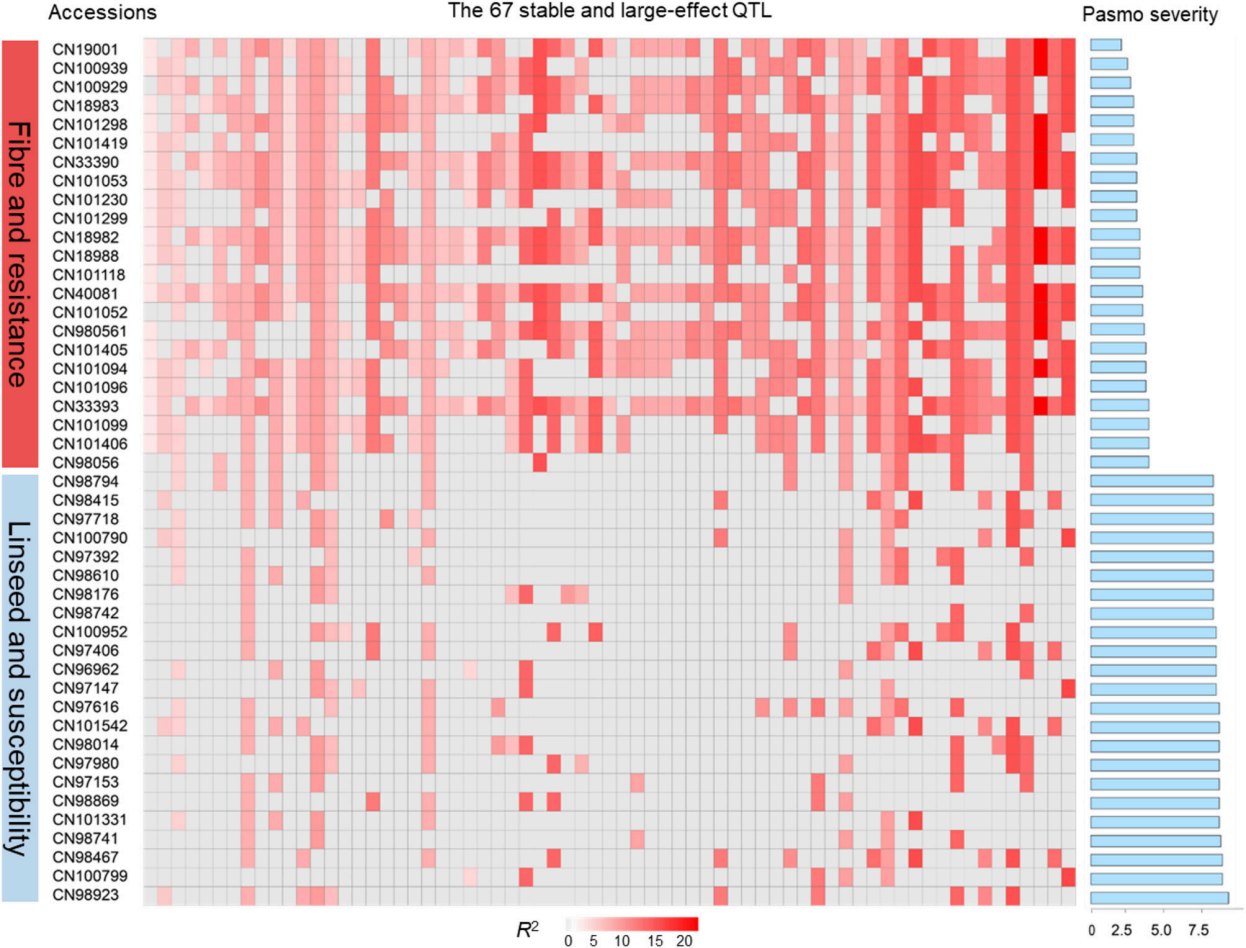
QTL genotypes, pasmo resistance and severity, and morphotypes of two extreme subsets representing 23 resistant and 23 susceptible accessions, respectively.

**Figure 4:**
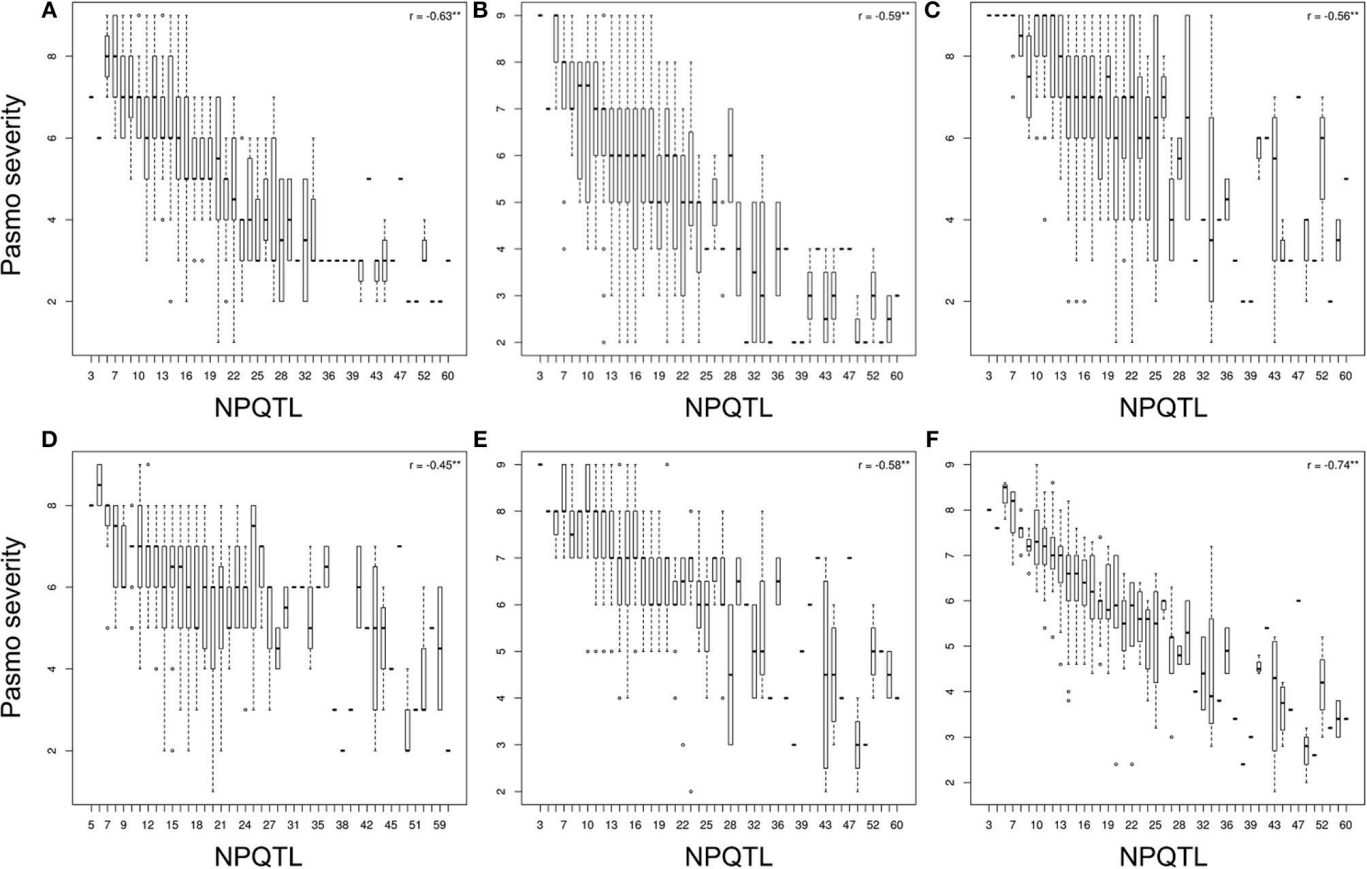
Box plots of the number of QTL with positive-effect alleles (NPQTL) in relation to pasmo severity during 2012-2016 **(A–E)** and average pasmo severity across 5 years **(F)**; ** indicates statistical significance at the 1% probability level.

The tally of the PQTL in the 370 accessions ranged from 3 to 60 per accession (Table S6). NPQTL were compared between two extreme subsets of 23 resistant (R) and 23 susceptible (S), respectively. Notably, all accessions of the R group belong to the fiber type and those of the S group were oilseed type. The R group, with an average PS rating of 3.2, contained an average of 42.5 PQTL per accession ranging from 14 to 60; the S group, with an average PS rating of 8.3, averaged only 9.4 PQTL per accession (Figure 3). Significant negative correlations between NPQTL and PS were observed in all six datasets (*r* = −0.45 to −0.74) (Figures 4A–F), with the highest negative correlation being with the mean PS rating dataset (*r* = −0.74) (Figure 4F).

### Association of PR and Its QTL With Flax Morphotype

**Figure 5:**
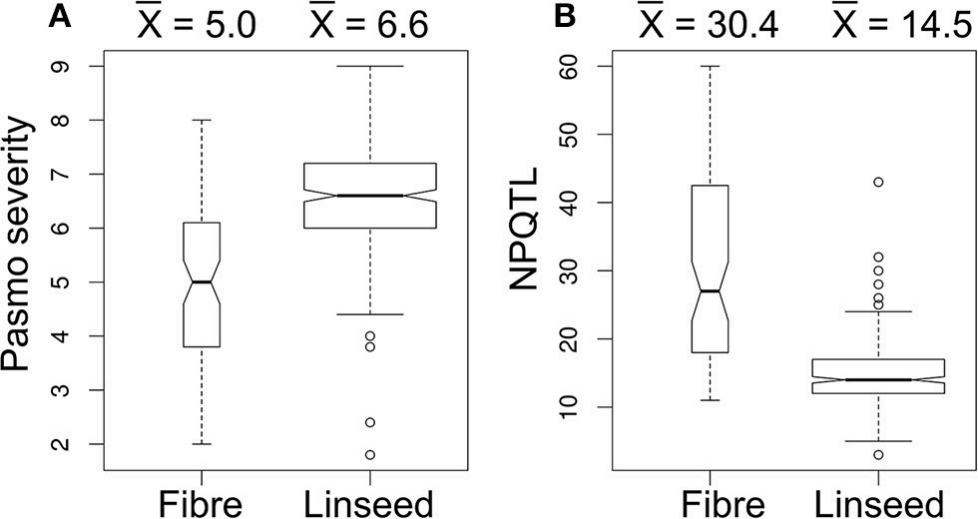
Box plots of pasmo severity **(A)** and the number of QTL with positive-effect alleles (NPQTL) **(B)** in relation to morphotypes.

A significant correlation between PS and morphotype (*r* = 0.49, *p* < 0.00001) was observed, showing that fiber accessions were more resistant to pasmo (Figure 5A). NPQTL were also significantly correlated with morphotypes (*r* = −0.65, *p* < 0.00001) (Figure 5B). Chi-square tests of independence were performed to identify PQTL alleles specifically belonging to a morphotype. For each QTL, the positive-effect allele was assigned a value of 0 and the alternate allele, a value of 1. Similarly, fiber type accessions were assigned 0 and linseed accessions 1. The chi-square test results indicated that most PQTL alleles were significantly associated with fiber type accessions (Table S7). For eight (8, 13, 14, 17, 21, 54, 55, and 63) of the 67 major QTL, between 80 and 100% of the PQTL were present in the fiber accessions; this was particularly acute for QTL 43 and 44 that were almost exclusive to the fiber germplasm. For the remaining 57 QTL, the PQTL were detected in fiber accessions (11–63 out of 80 fiber accessions) but were also found in many linseed accessions.

### Evaluation of the Flax Core Collection With QTL

**Figure 6:**
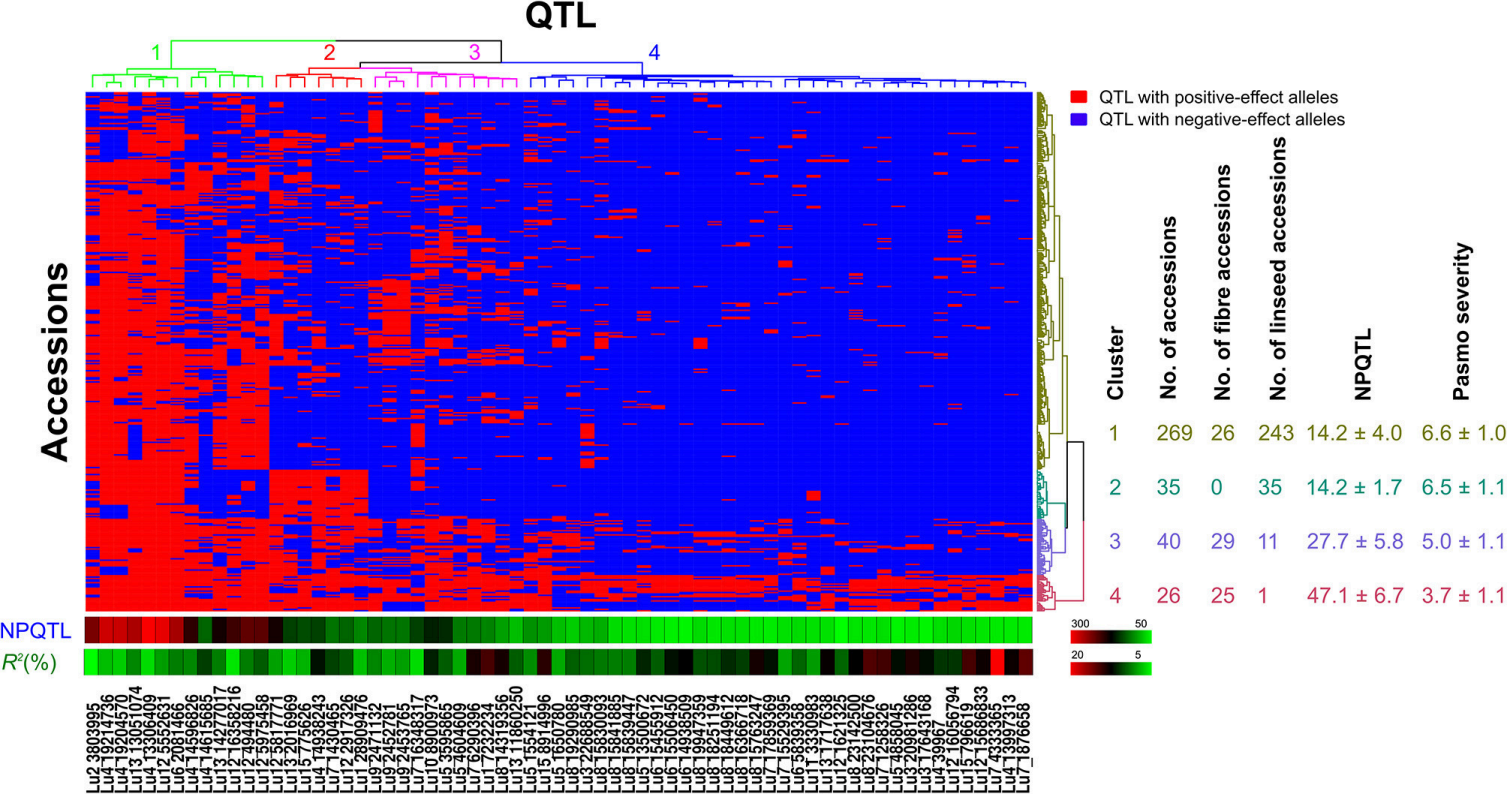
Cluster analysis of the association panel based on a set of 67 stable large-effect QTL. The accessions were grouped into four clusters and the QTL were assigned to four sub-groups. Tag QTNs of QTL were chosen for analysis. QTL with positive-effect alleles (PQTL) in the accessions are indicated in red; blue indicates the absence of PQTL. NPQTL, the number of QTL with positive-effect alleles.

Based on the 67 core QTL of the flax collection, bi-dimensional cluster analyses were conducted using tag QTNs as representatives of the QTL. The 370 accessions grouped into four clusters (Figure 6). Cluster 1 with 269 accessions and Cluster 2 with 35 were mostly susceptible to pasmo (PS ratings of 6.6 ± 1.0 and 6.5 ± 1.1, respectively). Most accessions (243) of Cluster 1 and all accessions of Cluster 2 were linseed type. Cluster 3 comprised 40 moderately susceptible (PS ratings of 5.0 ± 1.1) accessions including 11 of linseed. Cluster 4 contained 26 accessions, of which, 25 were fiber type and only one was a linseed; they were resistant to pasmo (PS ratings of 3.7 ± 1.1). The number of PQTL were 14.2 ± 4.0, 14.2 ± 1.7, 27.7 ± 5.8, and 47.1 ± 6.7 for Clusters 1–4, respectively. The 26 resistant accessions of Cluster 4 represent an important germplasm for PR breeding. This resistant germplasm included 14 of the 23 accessions in Figure 3 and CN101114, CN101115, CN101119, CN101237, CN101241, CN101296, CN101367, CN101395, CN101396, CN18987, CN98150, and CN98903.

The 67 QTL were clustered into four sub-groups. Group 1 included 13 QTL widely distributed across the germplasm (68.27% of the accessions) but with relatively low QTL effects (average *R*^2^ of 8.31%, ranging from 3.32 to 10.85%) (Figure 6; Table S7). Groups 2 and 3 contained 7 and 11 QTL, respectively. Present in 31.08% of the accessions, these QTL had an average *R*^2^ of 9.23%, ranging from 4.64 to 16.17%. The 36 QTL of Group 4 had an average *R*^2^ of 11.93%, ranging from 6.61 to 23.39% and contributing to the majority of the PR. These QTL were mostly found in the resistant accessions of Cluster 4 which amounts to a mere 9.70% of the germplasm. CN101367 with 43 QTL and CN19001 with 49 are good examples of resistant germplasm.

### Resistance Gene Analogs Co-localized With QTL

**Figure 7:**
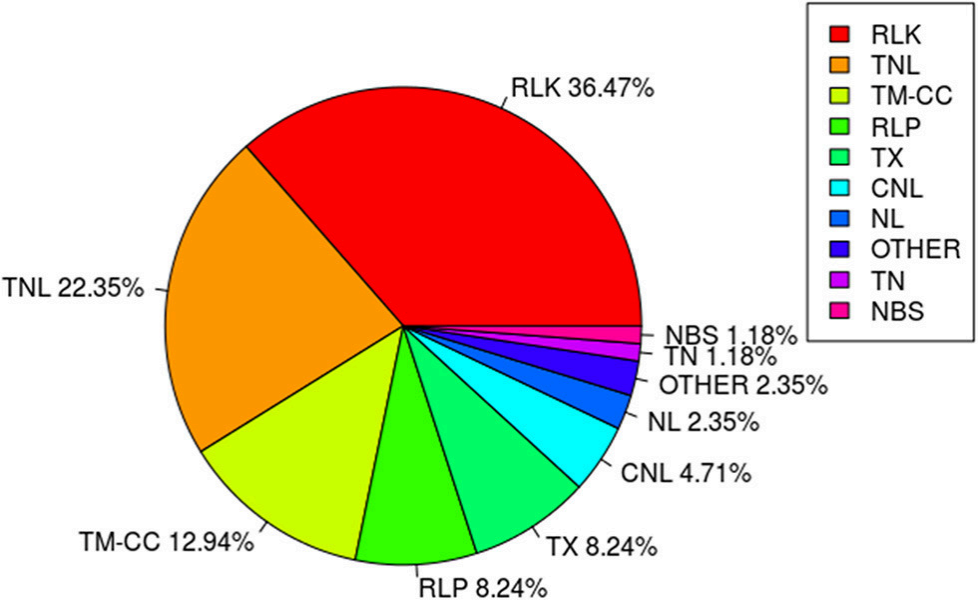
Class distribution of resistance gene analogs (RGAs) located within 200 Kb flanking regions of QTL.

Among the 67 stable and large-effect QTL, 45 co-localized with 85 RGAs within the pre-defined 200 Kb QTL flanking window. Four types of RGAs were harbored at these loci: receptor like protein (RLP), receptor like kinase (RLK), nucleotide-binding site (NBS) coding genes (including TNL, TX, CNL, NL, TN, NBS, and others), and transmembrane- coiled coil protein (TM-CC) (Sekhwal et al., 2015). The majority of the RGAs were RLKs with 36.47%, followed by TNLs with 22.35% (Figure 7), including a TNL cluster associated with QTL 37 and 38 on chromosome 8 (Table 8). Additional TNL-type RGA clusters co-localized with QTL 13 and 14 on chromosome 4 and QTL 15 and 16 on chromosome 5. An RLP gene (*Lus10039958*) located only 56 bp downstream of QTN Lu10-8700793 (QTL 49) exemplifies close linkage between the RGA and the QTL identified in this study. A TNL gene (*Lus10007812*) located 99 Kb downstream of QTN Lu8-18251174 (QTL 37) was the farthest RGA from its QTL.

## Discussion

### Pasmo Resistance in the Core Collection

Pasmo is widespread throughout all flax growing regions (Halley et al., 2004), but no flax cultivars are truly resistant to pasmo (Diederichsen et al., 2008). Evaluation of pasmo disease response revealed a range of resistance levels in the core collection (You et al., 2017) and, consistent with previous observations, no immune or highly resistant flax varieties were identified. However, some accessions displayed a relatively high level of resistance to pasmo and had high NPQTL (Figure 3; Table S1). For example, CN19001, a fiber type from the Netherlands, and CN101367, a linseed accession from Georgia, had respective 5-years average PS ratings of 2.0 and 1.8 and possessed 49 and 43 NPQTL, respectively. These accessions of fiber and linseed lineages are good parents for improvement of flax resistance through direct hybridization with elite varieties. Moderately resistant and moderately susceptible lines accounted for 6.49–21.35 and 20.81–42.16% of all accessions in the association panel depending on the years, respectively. Due to the quantitative nature of the disease, this germplasm also holds potential in breeding through the pyramiding of QTL with smaller effects, a strategy that has been successful in improving FHB resistance in wheat (Buerstmayr et al., 2009).

The fiber accessions were generally more resistant to pasmo than the linseed accessions, not surprisingly considering that fiber flax is cultivated for its stem fibers whose quality is greatly affected by the disease. From flowering to maturity, the dark brown to black bands that appear on the stems of infected plants can reduce the quality of the fiber (Colhoun and Muskett, 1943). The relatively higher level of resistance of the fiber type is likely a reflection of artificial selection and possibly independent domestication of the fiber flax lineage (Fu et al., 2012). The transfer of PR from fiber to linseed types can be considered, particularly in schemes where faster recovery of the recurrent linseed types can be achieved by marker-assisted backcrossing for example.

Pasmo resistance levels also varied significantly among genotypes from different geographical origins (You et al., 2017). Rainfall accumulation from June to August was significantly and positively associated with pasmo incidence and severity (Halley et al., 2004). Therefore, natural selection might be the main evolutionary pressure resulting in geographic variation. Accessions from India and Pakistan were the most susceptible of the core collection; this is not surprising considering that the environmental conditions of the flax growing regions of India are not conducive to the disease development (Diederichsen et al., 2008). On the other hand, accessions from Europe were the most resistant, a reflection of the fiber type predominance of the European germplasm (You et al., 2017) that have historically been under higher selection pressure for PR. North America appears to have the largest proportion of moderately susceptible and susceptible accessions (63 and 55) of the diversity panel, in agreement with its almost exclusive linseed germplasm (You et al., 2017). The most resistant Canadian linseed breeding line, CN101536, is only moderately resistant with an average PS of 4.4. Therefore, the incorporation of PR from linseed accession CN101367 from Georgia, as earlier noted, would benefit the improvement of PR in linseed. Interestingly, the East Asian mixed fiber and linseed germplasm is globally moderately resistant, in agreement with the long history of domestication for PR (Millam et al., 2005).

The PS of moderately resistant and susceptible accessions varied considerably across years, indicating a strong genotype × environment interaction. The low but significant correlations between the phenotypic data from any 2 years suggest the presence of interactions. In addition, the variance component analysis showed that the genotype × environment interaction accounted for a large proportion of the total variation. The interaction partially resulted in different QTL identified in datasets from different years. QTL contribution to PR might marginally differ from year to year, stressing the need for multi-environment phenotyping to identify environment-specific QTL.

### Methods Comparison

In this study, few QTL were detected by the single-locus methods MLM and GEMMA, likely as a consequence of the stringent corrected probability threshold (1.93 × 10^−7^); the third single-locus method GLM and the multi-locus methods identified greater numbers of QTL. The numbers of QTL identified by GLM and mrMLM methods were similar while FarmCPU detected comparatively fewer. The phenotypic variance explained by the QTL (*R*^2^) is also an important criterion of comparison. Both single and multi-locus methods identified some QTL with large effects (Table 4). However, most small-effect QTL were detected only when multi-locus methods were used. Although few QTL were common between methods, a large proportion of common QTL was observed among mrMLM methods. Thus, the complementarity between different methods is significant, and, in light of our results, the combined utilization of various statistical models is highly recommended for the identification of all potential QTL with both large and small effects.

### Evaluation of Pasmo QTL in the Core Collection and Breeding Applications

Identification of QTL associated with PR can potentially facilitate their incorporation into elite germplasm, especially in North America where linseed is the main type for production (You et al., 2017). Several large-effect QTL/tag QTNs were noted, including QTL 44/Lu9-4333365 (*R*^2^ = 23.39%), QTL 43/Lu9-1896658 (*R*^2^ = 17.12%), QTL 39/Lu8-23104696 (*R*^2^ = 16.53%), and QTL 1/Lu1-9232234 (*R*^2^ = 16.17%). These were mostly present in resistant accessions as PQTL (Table 8) and they hold potential for MAS.

Although the large-effect QTL may be useful for MAS, a large number of small-effect QTL would be beneficial for genomic prediction (GP). GP using genome-wide markers to predict breeding values of target traits is a promising alternative method to MAS for low heritability traits including PR (Lipka et al., 2015; Poland and Rutkoski, 2016). Compared to conventional phenotypic selection, GP can accelerate genetic gains for early selection (Newell and Jannink, 2014). The accuracy and efficiency of GP models for flax PS were evaluated with three sets of QTL: 500 (SNP-500QTL), 134 (SNP-134QTL), and 67 (SNP-67QTL) which are developed in this study (He et al., 2018). The GP model built with SNP-500QTL achieved a prediction accuracy of 0.92 while the use of 134 and 67 QTL yielded accuracies of 0.75 and 0.76, respectively (He et al., 2018). The similar accuracies of the two smaller sets were expected because SNP-67QTL is essentially a non-redundant set of SNP-134QTL. These predictions serve as additional validation of the QTL identified herein and simultaneously illustrate the effectiveness of prediction models that include a full complement of large and small-effect QTL including environment-specific QTL.

### Candidate Genes for Pasmo Resistance

Functional annotation of the QTL identified herein revealed 85 RGAs co-located with 45 large-effect QTL. Of them, two RGAs, *Lus10031043*/RLK and *Lus10020016*/CNL corresponding to QTL 45/Lu9-6270375 and QTL 51/Lu12-474480, respectively, may be associated with two orthologous resistance genes in Arabidopsis (Xiang et al., 2008; Saijo et al., 2009). The RLK gene *Lus10031043* is an ortholog to *AT5G20480.1* in Arabidopsis, which encodes a predicted leucine-rich repeat receptor kinase (LRR-RLK) and functions as the receptor for bacterial pathogen-associated molecular patterns (PAMPs) EF-Tu (EFR). The LRR-RLK EFR recognizes the bacterial epitopes elf18, derived from elongation factor-Tu, and triggers the plant's immune response (Saijo et al., 2009). The *Pseudomonas syringae* effector *AvrPto* is reported to bind receptor kinases, including Arabidopsis EFR (LRR-RLK EFR), to inhibit plant PAMP-triggered immunity and, to subsequently trigger strong immune responses (Xiang et al., 2008). The flax CNL gene *Lus10020016* (*RPM1*) is orthologous to *RPM1* (*AT3G07040.1*) in Arabidopsis. *RPM1* contains an N-terminal tripartite nucleotide binding site and a C-terminal tandem array of leucine-rich repeats and confers resistance to *P. syringae* strains that carry the avirulence genes *avrB* and *avrRpm1* (https://www.arabidopsis.org/). The *RPM1* gene enables dual specificity to pathogens expressing either of two unrelated *P. syringae* avirulence genes (Grant et al., 1995). The above findings hint at *Lus10031043* and *Lus10020016* as potential candidate genes for PR.

## Conclusion

Using 10 statistical methods, a total of 500 QTL, including 67 stable and large-effect QTL and many additional small effect and environment-specific QTL were identified for PS, using a diversity panel of 370 flax accessions genotyped with 258,873 genome-wide SNPs and phenotyped in the field during 5 consecutive years. The large number of QTL identified in this study illustrates the complex genetic basis for PR in flax through a demonstration of its quantitative genetic nature and its sensitivity to environments. Multi-locus methods were able to detect small-effect QTL whereas the single-locus methods tended to identify fewer QTL of large effect. Various statistical methods identified mainly different sets of QTL, indicating the value of employing different statistical methods and multiple environment phenotypic data to capture the most comprehensive set of QTL: large, small and environment-specific. Combined utilization of multiple statistical methods is advantageous to identify QTL with small effects for traits with a complex genetic base and low heritability. The high correlation observed between PS and flax morphotype indicated that the fiber germplasm contains most of the PS QTL and constitutes an important genetic resource for flax PR breeding. The 67 large-effect QTL have potential in MAS and the 500 QTL set can be exploited for effective GP for improving pasmo resistance.

## Author Contributions

FY, SC, and KR conceived and designed the study. KR implemented the phenotyping of pasmo resistance. SC performed next-generation sequencing. FY conducted SNP identification and analysis. FY, LH, SC, ZY, PL, JX, and XW performed data analysis. LH, FY, and ZY prepared tables and figures. FY, LH, and JX drafted the manuscript. All authors reviewed and edited the manuscript.

### Conflict of Interest Statement

The authors declare that the research was conducted in the absence of any commercial or financial relationships that could be construed as a potential conflict of interest.
