# Supplementary material for: Genome-Wide Association Studies for Pasmo Resistance in Flax (Linum usitatissimum L.)
Source: Front Plant Sci. 2019 Jan 14;9:1982. doi: 10.3389/fpls.2018.01982 (PMC6339956; doi:10.3389/fpls.2018.01982)
Supplement: Supplementary file 2 [file Table_2.DOCX]

**Supplementary table**

**Table S2** Basic **s**tatistics of pasmo severity across five years

| **Year/ assessment stage** | **Mean** | **Min** | **Max** | **Median** | **Std** | **CV** |
| --- | --- | --- | --- | --- | --- | --- |
| 2012/P1 | 3.00 | 1.00 | 6.00 | 3.00 | 1.00 | 0.33 |
| 2012/P2 | 3.74 | 1.00 | 8.00 | 4.00 | 1.42 | 0.38 |
| 2012/P3 | 4.48 | 1.00 | 9.00 | 4.00 | 1.71 | 0.38 |
| 2012/P4 | 5.57 | 1.00 | 9.00 | 6.00 | 1.86 | 0.33 |
| 2013/P1 | 2.69 | 1.00 | 8.00 | 2.00 | 1.31 | 0.49 |
| 2013/P2 | 3.79 | 1.00 | 8.00 | 4.00 | 1.59 | 0.42 |
| 2013/P3 | 4.60 | 1.00 | 9.00 | 5.00 | 1.76 | 0.38 |
| 2013/P4 | 5.69 | 2.00 | 9.00 | 6.00 | 1.91 | 0.34 |
| 2014/P1 | 2.56 | 1.00 | 6.00 | 2.00 | 1.29 | 0.50 |
| 2014/P2 | 6.04 | 1.00 | 9.00 | 6.00 | 2.18 | 0.36 |
| 2014/P3 | 6.86 | 1.00 | 9.00 | 7.00 | 2.07 | 0.30 |
| 2015/P1 | 2.32 | 1.00 | 6.00 | 2.00 | 1.23 | 0.53 |
| 2015/P2 | 4.90 | 1.00 | 8.00 | 5.00 | 1.52 | 0.31 |
| 2015/P3 | 6.08 | 1.00 | 9.00 | 6.00 | 1.58 | 0.26 |
| 2016/P1 | 3.31 | 1.00 | 8.00 | 3.00 | 1.42 | 0.43 |
| 2016/P2 | 4.96 | 1.00 | 9.00 | 5.00 | 1.51 | 0.30 |
| 2016/P3 | 5.82 | 1.00 | 9.00 | 6.00 | 1.55 | 0.27 |
| 2016/P4 | 6.72 | 2.00 | 9.00 | 7.00 | 1.37 | 0.20 |
| Mean | 6.22 | 1.80 | 9.00 | 6.40 | 1.32 | 0.21 |

Std: standard deviation; CV: coefficient of variation; Mean: the average pasmo severity of five years’ data
