# Supplementary material for: Genome-Wide Association Studies for Pasmo Resistance in Flax (Linum usitatissimum L.)
Source: Front Plant Sci. 2019 Jan 14;9:1982. doi: 10.3389/fpls.2018.01982 (PMC6339956; doi:10.3389/fpls.2018.01982)
Supplement: Supplementary file 3 [file Table_3.DOCX]

**Supplementary table**

**Table S3** Variance component analysis of pasmo severity across five years (2012-2016)

| **Name** | **Variance** | **Std** | **Percentage** |
| --- | --- | --- | --- |
| Accession | 1.41 | 1.19 | 41.18 |
| Year | 0.33 | 0.58 | 9.65 |
| Accession × Year | 0.83 | 0.91 | 24.23 |
| Residual | 0.85 | 0.92 | 24.94 |

Std: standard deviation
