# Supplementary material for: Genome-Wide Association Studies for Pasmo Resistance in Flax (Linum usitatissimum L.)
Source: Front Plant Sci. 2019 Jan 14;9:1982. doi: 10.3389/fpls.2018.01982 (PMC6339956; doi:10.3389/fpls.2018.01982)
Supplement: Supplementary file 5 [file Table_5.DOCX]

**Supplementary table**

**Table S5** Tag QTNs identified by two or more statistical methods.

| **QTL No.** | **QTN** | **Chr** | **Pos** | **SNP** | ***R*^2^ (%)** | **Statistical models** | **No of statistical models** | **Datasets** | **No of datasets** |
| --- | --- | --- | --- | --- | --- | --- | --- | --- | --- |
| 441 | Lu13-15732180 | 13 | 15732180 | A/C | 3.59 | ISIS EM-BLASSO, GEMMA, FASTmrMLM, pKWmEB, mrMLM, GLM | 6 | 2014 | 1 |
| 251 | Lu8-17237505 | 8 | 17237505 | G/T | 6.62 | ISIS EM-BLASSO, FASTmrEMMA, GLM, pLARmEB, mrMLM | 5 | mean, 2014, 2016, 2012 | 4 |
| 343 | Lu11-4144862 | 11 | 4144862 | T/C | 12.68 | GEMMA, ISIS EM-BLASSO, pKWmEB, GLM, pLARmEB | 5 | 2014, mean, 2016, 2013 | 4 |
| 428 | Lu13-14282050 | 13 | 14282050 | C/T | 2.03 | pKWmEB, GLM, pLARmEB, FASTmrMLM | 4 | 2013, mean | 2 |
| 157 | Lu5-469937 | 5 | 469937 | G/C | 1.6 | FASTmrMLM, pKWmEB, mrMLM, pLARmEB | 4 | 2015 | 1 |
| 197 | Lu6-17054363 | 6 | 17054363 | T/G | 4.49 | pLARmEB, mrMLM, FASTmrEMMA, FASTmrMLM | 4 | 2013 | 1 |
| 251 | Lu8-17270785 | 8 | 17270785 | C/G | 6.37 | pKWmEB, GLM, FASTmrMLM | 3 | 2012, mean, 2014 | 3 |
| 479 | Lu15-974597 | 15 | 974597 | G/C | 11.41 | GLM, mrMLM, FarmCPU | 3 | mean, 2016, 2015 | 3 |
| 142 | Lu4-14758081 | 4 | 14758081 | G/T | 3.92 | FASTmrMLM, GLM, pKWmEB | 3 | 2014, mean | 2 |
| 280 | Lu9-1414306 | 9 | 1414306 | G/A | 13.61 | GEMMA, GLM, mrMLM | 3 | 2016, mean | 2 |
| 480 | Lu15-988037 | 15 | 988037 | T/G | 6.23 | GLM, pKWmEB, ISIS EM-BLASSO | 3 | mean, 2015 | 2 |
| 77 | Lu3-4694214 | 3 | 4694214 | C/T | 1.55 | FASTmrEMMA, pKWmEB, ISIS EM-BLASSO | 3 | 2015 | 1 |
| 189 | Lu6-9154179 | 6 | 9154179 | G/A | 3.21 | FASTmrMLM, ISIS EM-BLASSO, pKWmEB | 3 | 2013 | 1 |
| 259 | Lu8-20031761 | 8 | 20031761 | A/G | 8.21 | GLM, pLARmEB, ISIS EM-BLASSO | 3 | mean | 1 |
| 358 | Lu12-44912 | 12 | 44912 | G/A | 3.74 | FASTmrMLM, FASTmrEMMA, pLARmEB | 3 | 2012 | 1 |
| 364 | Lu12-1196812 | 12 | 1196812 | C/T | 4.96 | pLARmEB, pKWmEB, FASTmrMLM | 3 | 2015 | 1 |
| 444 | Lu13-17062842 | 13 | 17062842 | G/A | 0.25 | FASTmrMLM, pKWmEB, FASTmrEMMA | 3 | 2012 | 1 |
| 482 | Lu15-1002029 | 15 | 1002029 | T/C | 5.27 | FASTmrMLM, pKWmEB, pLARmEB | 3 | 2016 | 1 |
| 143 | Lu4-14810782 | 4 | 14810782 | C/A | 4.65 | ISIS EM-BLASSO, GLM | 2 | 2012, mean, 2016 | 3 |
| 243 | Lu8-15841727 | 8 | 15841727 | C/T | 9.18 | ISIS EM-BLASSO, GLM | 2 | 2016, mean, 2012 | 3 |
| 442 | Lu13-15803055 | 13 | 15803055 | G/T | 1.29 | mrMLM, GLM | 2 | mean, 2012, 2015 | 3 |
| 63 | Lu2-22813337 | 2 | 22813337 | G/T | 2.59 | GLM, FASTmrEMMA | 2 | 2016, mean | 2 |
| 143 | Lu4-14810573 | 4 | 14810573 | G/T | 5.05 | GLM, FASTmrMLM | 2 | 2016, mean | 2 |
| 225 | Lu8-3209265 | 8 | 3209265 | G/A | 8.48 | pKWmEB, FASTmrMLM | 2 | mean, 2013 | 2 |
| 233 | Lu8-14331564 | 8 | 14331564 | T/C | 3.18 | GLM, FASTmrMLM | 2 | mean, 2015 | 2 |
| 250 | Lu8-16904194 | 8 | 16904194 | G/A | 9.09 | GLM, ISIS EM-BLASSO | 2 | mean, 2014 | 2 |
| 330 | Lu10-16153439 | 10 | 16153439 | C/T | 1.4 | FASTmrMLM, pLARmEB | 2 | 2015, mean | 2 |
| 375 | Lu12-5578883 | 12 | 5578883 | G/A | 7.14 | FarmCPU, GLM | 2 | 2013, mean | 2 |
| 5 | Lu1-4886034 | 1 | 4886034 | C/T | 2.33 | GLM, FarmCPU | 2 | 2015 | 1 |
| 31 | Lu1-17921907 | 1 | 17921907 | A/G | 0.67 | mrMLM, ISIS EM-BLASSO | 2 | 2013 | 1 |
| 42 | Lu1-28931153 | 1 | 28931153 | A/G | 0.89 | pLARmEB, pKWmEB | 2 | 2016 | 1 |
| 77 | Lu3-4694633 | 3 | 4694633 | C/T | 1.91 | FASTmrMLM, pLARmEB | 2 | 2015 | 1 |
| 173 | Lu5-10415458 | 5 | 10415458 | C/T | 1.35 | pLARmEB, FASTmrMLM | 2 | 2016 | 1 |
| 258 | Lu8-19992045 | 8 | 19992045 | A/G | 5.37 | FASTmrMLM, pLARmEB | 2 | 2012 | 1 |
| 259 | Lu8-20030216 | 8 | 20030216 | A/G | 7.34 | GLM, FASTmrMLM | 2 | mean | 1 |
| 385 | Lu12-15759574 | 12 | 15759574 | C/G | 3.04 | pKWmEB, GLM | 2 | mean | 1 |
| 415 | Lu13-3720348 | 13 | 3720348 | C/G | 1.7 | FASTmrEMMA, mrMLM | 2 | 2015 | 1 |
| 431 | Lu13-14952991 | 13 | 14952991 | A/T | 6.07 | ISIS EM-BLASSO, FASTmrEMMA | 2 | 2012 | 1 |
| 444 | Lu13-17063922 | 13 | 17063922 | T/A | 0.72 | pLARmEB, mrMLM | 2 | 2012 | 1 |
| 132 | Lu4-14608434 | 4 | 14608434 | A/C | 7.79 | GLM | 1 | mean, 2014, 2012 | 3 |
| 267 | Lu8-22902192 | 8 | 22902192 | A/C | 11.5 | GLM | 1 | mean, 2014, 2012 | 3 |
| 407 | Lu13-2011686 | 13 | 2011686 | T/C | 8.04 | GLM | 1 | 2016, mean, 2014 | 3 |
| 479 | Lu15-972417 | 15 | 972417 | G/A | 10.5 | GLM | 1 | 2015, 2016, mean | 3 |
| 18 | Lu1-7750311 | 1 | 7750311 | A/T | 7.09 | GLM | 1 | mean, 2014 | 2 |
| 18 | Lu1-7750388 | 1 | 7750388 | C/G | 8.12 | GLM | 1 | 2016, mean | 2 |
| 18 | Lu1-7751552 | 1 | 7751552 | A/G | 6.38 | GLM | 1 | 2014, mean | 2 |
| 19 | Lu1-7752867 | 1 | 7752867 | A/G | 3.82 | GLM | 1 | 2014, mean | 2 |
| 19 | Lu1-7752950 | 1 | 7752950 | A/T | 4.61 | GLM | 1 | 2014, mean | 2 |
| 23 | Lu1-9140757 | 1 | 9140757 | T/C | 12.11 | GLM | 1 | 2012, mean | 2 |
| 45 | Lu2-1670668 | 2 | 1670668 | C/T | 3.19 | GLM | 1 | 2016, mean | 2 |
| 117 | Lu4-13816083 | 4 | 13816083 | T/C | 9.94 | GLM | 1 | 2016, mean | 2 |
| 132 | Lu4-14609120 | 4 | 14609120 | A/T | 7.38 | GLM | 1 | mean, 2014 | 2 |
| 138 | Lu4-14620844 | 4 | 14620844 | C/T | 3.04 | GLM | 1 | mean, 2014 | 2 |
| 140 | Lu4-14722131 | 4 | 14722131 | A/C | 10.97 | GLM | 1 | mean, 2013 | 2 |
| 143 | Lu4-14804680 | 4 | 14804680 | C/A | 3.63 | GLM | 1 | 2014, mean | 2 |
| 143 | Lu4-14808640 | 4 | 14808640 | A/G | 5.02 | GLM | 1 | 2016, mean | 2 |
| 143 | Lu4-14809348 | 4 | 14809348 | C/T | 4.91 | GLM | 1 | mean, 2016 | 2 |
| 145 | Lu4-14822805 | 4 | 14822805 | T/G | 2.42 | GLM | 1 | mean, 2012 | 2 |
| 146 | Lu4-14857578 | 4 | 14857578 | A/G | 5.49 | GLM | 1 | 2016, mean | 2 |
| 186 | Lu6-5397940 | 6 | 5397940 | G/A | 4.63 | GLM | 1 | 2014, mean | 2 |
| 186 | Lu6-5428245 | 6 | 5428245 | C/T | 4.6 | GLM | 1 | mean, 2014 | 2 |
| 249 | Lu8-16331798 | 8 | 16331798 | C/G | 10.46 | GLM | 1 | 2016, mean | 2 |
| 250 | Lu8-16646597 | 8 | 16646597 | C/T | 10.11 | GLM | 1 | mean, 2014 | 2 |
| 250 | Lu8-16694508 | 8 | 16694508 | C/T | 9.15 | GLM | 1 | 2014, mean | 2 |
| 250 | Lu8-17230253 | 8 | 17230253 | C/T | 6.73 | GLM | 1 | mean, 2012 | 2 |
| 253 | Lu8-18154338 | 8 | 18154338 | C/A | 7.34 | GLM | 1 | mean, 2012 | 2 |
| 258 | Lu8-20000197 | 8 | 20000197 | C/G | 5.35 | GLM | 1 | 2014, mean | 2 |
| 259 | Lu8-20077050 | 8 | 20077050 | A/G | 8.82 | GLM | 1 | mean, 2014 | 2 |
| 270 | Lu8-23103891 | 8 | 23103891 | G/A | 8.35 | GLM | 1 | mean, 2012 | 2 |
| 309 | Lu9-19999159 | 9 | 19999159 | C/T | 1.8 | GLM | 1 | 2013, mean | 2 |
| 338 | Lu11-2947405 | 11 | 2947405 | A/G | 4.76 | GLM | 1 | mean, 2016 | 2 |
| 375 | Lu12-5570351 | 12 | 5570351 | T/C | 7.56 | GLM | 1 | mean, 2013 | 2 |
| 375 | Lu12-5573010 | 12 | 5573010 | G/A | 7.67 | GLM | 1 | 2013, mean | 2 |
| 387 | Lu12-15789586 | 12 | 15789586 | C/T | 1.73 | GLM | 1 | 2012, mean | 2 |
| 387 | Lu12-15789680 | 12 | 15789680 | C/T | 1.84 | GLM | 1 | mean, 2012 | 2 |
| 397 | Lu13-1915532 | 13 | 1915532 | C/G | 6.17 | GLM | 1 | mean, 2014 | 2 |
| 485 | Lu15-3662277 | 15 | 3662277 | G/C | 8.78 | GLM | 1 | 2016, mean | 2 |
| 5 | Lu1-4874616 | 1 | 4874616 | A/G | 1.52 | pKWmEB | 1 | 2013 | 1 |
| 7 | Lu1-5150780 | 1 | 5150780 | T/C | 1.5 | ISIS EM-BLASSO | 1 | 2014 | 1 |
| 11 | Lu1-6245350 | 1 | 6245350 | T/A | 2.16 | pKWmEB | 1 | 2014 | 1 |
| 18 | Lu1-7750045 | 1 | 7750045 | A/G | 7.23 | GLM | 1 | mean | 1 |
| 18 | Lu1-7750175 | 1 | 7750175 | G/T | 8.9 | GLM | 1 | mean | 1 |
| 22 | Lu1-8785558 | 1 | 8785558 | T/A | 2.12 | FASTmrMLM | 1 | 2016 | 1 |
| 23 | Lu1-9120437 | 1 | 9120437 | G/A | 10.63 | GLM | 1 | mean | 1 |
| 23 | Lu1-9130861 | 1 | 9130861 | A/G | 10.14 | GLM | 1 | mean | 1 |
| 39 | Lu1-28776031 | 1 | 28776031 | A/G | 4.86 | GLM | 1 | mean | 1 |
| 39 | Lu1-28783666 | 1 | 28783666 | G/T | 3.96 | GLM | 1 | 2014 | 1 |
| 51 | Lu2-3803078 | 2 | 3803078 | C/T | 1.98 | pKWmEB | 1 | mean | 1 |
| 87 | Lu3-18559066 | 3 | 18559066 | C/T | 3.29 | mrMLM | 1 | 2014 | 1 |
| 100 | Lu4-381329 | 4 | 381329 | C/T | 0.42 | mrMLM | 1 | 2012 | 1 |
| 100 | Lu4-381392 | 4 | 381392 | A/G | 0.45 | ISIS EM-BLASSO | 1 | 2013 | 1 |
| 115 | Lu4-13702763 | 4 | 13702763 | A/G | 1.4 | GLM | 1 | mean | 1 |
| 116 | Lu4-13790860 | 4 | 13790860 | T/C | 10.03 | GLM | 1 | mean | 1 |
| 117 | Lu4-13815797 | 4 | 13815797 | C/T | 9.06 | GLM | 1 | mean | 1 |
| 118 | Lu4-14146207 | 4 | 14146207 | C/A | 5.83 | GLM | 1 | mean | 1 |
| 120 | Lu4-14210011 | 4 | 14210011 | G/T | 9.62 | GLM | 1 | mean | 1 |
| 123 | Lu4-14284967 | 4 | 14284967 | C/T | 2.52 | GLM | 1 | mean | 1 |
| 123 | Lu4-14287664 | 4 | 14287664 | A/G | 2.63 | GLM | 1 | mean | 1 |
| 123 | Lu4-14289303 | 4 | 14289303 | G/C | 2.3 | GLM | 1 | mean | 1 |
| 123 | Lu4-14297669 | 4 | 14297669 | C/T | 2.7 | GLM | 1 | mean | 1 |
| 127 | Lu4-14444424 | 4 | 14444424 | G/C | 2.39 | GLM | 1 | mean | 1 |
| 131 | Lu4-14606969 | 4 | 14606969 | A/T | 5.63 | GLM | 1 | mean | 1 |
| 136 | Lu4-14611600 | 4 | 14611600 | C/T | 6.59 | GLM | 1 | 2014 | 1 |
| 138 | Lu4-14620715 | 4 | 14620715 | G/T | 3.41 | GLM | 1 | 2012 | 1 |
| 138 | Lu4-14620756 | 4 | 14620756 | A/G | 5.58 | GLM | 1 | 2012 | 1 |
| 139 | Lu4-14637101 | 4 | 14637101 | T/C | 1.62 | GLM | 1 | mean | 1 |
| 141 | Lu4-14737622 | 4 | 14737622 | G/A | 5.89 | GLM | 1 | mean | 1 |
| 143 | Lu4-14811019 | 4 | 14811019 | A/C | 4.02 | GLM | 1 | mean | 1 |
| 145 | Lu4-14814992 | 4 | 14814992 | T/C | 2.24 | GLM | 1 | mean | 1 |
| 146 | Lu4-14850494 | 4 | 14850494 | T/C | 4.79 | GLM | 1 | mean | 1 |
| 146 | Lu4-14877833 | 4 | 14877833 | A/C | 6.19 | pKWmEB | 1 | 2015 | 1 |
| 148 | Lu4-15096801 | 4 | 15096801 | T/C | 1.69 | GLM | 1 | 2012 | 1 |
| 170 | Lu5-4848063 | 5 | 4848063 | A/G | 8.19 | GLM | 1 | mean | 1 |
| 172 | Lu5-10360045 | 5 | 10360045 | C/G | 2.14 | ISIS EM-BLASSO | 1 | mean | 1 |
| 182 | Lu6-2415314 | 6 | 2415314 | A/G | 5.32 | GLM | 1 | 2012 | 1 |
| 182 | Lu6-2417796 | 6 | 2417796 | A/T | 6.01 | GLM | 1 | 2012 | 1 |
| 188 | Lu6-6004425 | 6 | 6004425 | C/T | 2.56 | pLARmEB | 1 | 2013 | 1 |
| 190 | Lu6-9867044 | 6 | 9867044 | C/G | 6.36 | GLM | 1 | mean | 1 |
| 194 | Lu6-15506329 | 6 | 15506329 | C/T | 7.86 | GLM | 1 | mean | 1 |
| 195 | Lu6-16415796 | 6 | 16415796 | C/T | 1.69 | GLM | 1 | mean | 1 |
| 199 | Lu7-2452149 | 7 | 2452149 | C/T | 1.75 | GLM | 1 | mean | 1 |
| 208 | Lu7-4237735 | 7 | 4237735 | G/T | 1.2 | ISIS EM-BLASSO | 1 | mean | 1 |
| 211 | Lu7-5223437 | 7 | 5223437 | C/T | 1.37 | GLM | 1 | 2012 | 1 |
| 225 | Lu8-3225025 | 8 | 3225025 | T/A | 6.6 | pLARmEB | 1 | 2013 | 1 |
| 233 | Lu8-14340718 | 8 | 14340718 | G/A | 2.82 | GLM | 1 | mean | 1 |
| 235 | Lu8-15457893 | 8 | 15457893 | T/C | 7.33 | GLM | 1 | 2016 | 1 |
| 243 | Lu8-15841885 | 8 | 15841885 | T/C | 5.4 | GLM | 1 | mean | 1 |
| 246 | Lu8-15963010 | 8 | 15963010 | A/T | 7.16 | GLM | 1 | mean | 1 |
| 246 | Lu8-15963150 | 8 | 15963150 | G/T | 7.61 | GLM | 1 | mean | 1 |
| 249 | Lu8-16366918 | 8 | 16366918 | C/T | 7.13 | GLM | 1 | mean | 1 |
| 253 | Lu8-18152249 | 8 | 18152249 | A/G | 5.44 | GLM | 1 | mean | 1 |
| 253 | Lu8-18152847 | 8 | 18152847 | C/G | 5.11 | GLM | 1 | mean | 1 |
| 258 | Lu8-19992872 | 8 | 19992872 | A/G | 5.03 | GLM | 1 | 2014 | 1 |
| 258 | Lu8-19996289 | 8 | 19996289 | A/C | 5.16 | GLM | 1 | 2014 | 1 |
| 259 | Lu8-20031082 | 8 | 20031082 | C/T | 7.7 | GLM | 1 | mean | 1 |
| 259 | Lu8-20031626 | 8 | 20031626 | T/C | 6.67 | GLM | 1 | mean | 1 |
| 259 | Lu8-20031882 | 8 | 20031882 | C/T | 8.18 | GLM | 1 | mean | 1 |
| 259 | Lu8-20076947 | 8 | 20076947 | A/G | 8.45 | GLM | 1 | 2014 | 1 |
| 267 | Lu8-22907525 | 8 | 22907525 | A/T | 3.4 | FASTmrEMMA | 1 | 2014 | 1 |
| 278 | Lu9-1217851 | 9 | 1217851 | G/A | 0.88 | FASTmrEMMA | 1 | 2014 | 1 |
| 279 | Lu9-1249332 | 9 | 1249332 | G/A | 9.1 | GLM | 1 | mean | 1 |
| 279 | Lu9-1250164 | 9 | 1250164 | G/A | 9.5 | GLM | 1 | mean | 1 |
| 280 | Lu9-1430465 | 9 | 1430465 | G/C | 7.37 | GLM | 1 | 2016 | 1 |
| 281 | Lu9-1436323 | 9 | 1436323 | T/C | 0.29 | ISIS EM-BLASSO | 1 | 2015 | 1 |
| 283 | Lu9-2126112 | 9 | 2126112 | A/G | 1.64 | pLARmEB | 1 | 2016 | 1 |
| 287 | Lu9-3999527 | 9 | 3999527 | G/A | 5.95 | GLM | 1 | 2013 | 1 |
| 293 | Lu9-5595092 | 9 | 5595092 | A/G | 10.83 | GLM | 1 | mean | 1 |
| 293 | Lu9-5907414 | 9 | 5907414 | G/T | 5.67 | mrMLM | 1 | 2012 | 1 |
| 297 | Lu9-12304004 | 9 | 12304004 | A/G | 2.5 | ISIS EM-BLASSO | 1 | 2013 | 1 |
| 298 | Lu9-15301547 | 9 | 15301547 | A/G | 2.21 | mrMLM | 1 | mean | 1 |
| 300 | Lu9-16100896 | 9 | 16100896 | G/A | 2.86 | mrMLM | 1 | 2012 | 1 |
| 305 | Lu9-19456547 | 9 | 19456547 | T/G | 2.16 | pKWmEB | 1 | 2013 | 1 |
| 307 | Lu9-19855693 | 9 | 19855693 | G/A | 7.78 | GLM | 1 | mean | 1 |
| 308 | Lu9-19957506 | 9 | 19957506 | G/A | 1.36 | GLM | 1 | mean | 1 |
| 308 | Lu9-19965692 | 9 | 19965692 | C/T | 1.33 | GLM | 1 | mean | 1 |
| 332 | Lu10-16903790 | 10 | 16903790 | C/T | 1.42 | GLM | 1 | mean | 1 |
| 346 | Lu11-4339807 | 11 | 4339807 | G/C | 7.57 | GLM | 1 | mean | 1 |
| 347 | Lu11-4407386 | 11 | 4407386 | G/A | 8.46 | GLM | 1 | 2014 | 1 |
| 348 | Lu11-5976511 | 11 | 5976511 | A/T | 10.18 | GLM | 1 | mean | 1 |
| 367 | Lu12-1873664 | 12 | 1873664 | T/C | 3.12 | FASTmrEMMA | 1 | 2012 | 1 |
| 369 | Lu12-2548498 | 12 | 2548498 | T/C | 0.9 | mrMLM | 1 | mean | 1 |
| 370 | Lu12-2724279 | 12 | 2724279 | G/A | 11.05 | GLM | 1 | mean | 1 |
| 375 | Lu12-5561961 | 12 | 5561961 | T/C | 6.52 | GLM | 1 | mean | 1 |
| 375 | Lu12-5568270 | 12 | 5568270 | C/A | 6.14 | GLM | 1 | mean | 1 |
| 375 | Lu12-5570067 | 12 | 5570067 | T/C | 7.1 | GLM | 1 | mean | 1 |
| 375 | Lu12-5580187 | 12 | 5580187 | G/A | 5.46 | GLM | 1 | mean | 1 |
| 381 | Lu12-6549664 | 12 | 6549664 | C/G | 1.5 | FASTmrEMMA | 1 | 2015 | 1 |
| 385 | Lu12-15758393 | 12 | 15758393 | G/A | 2.44 | GLM | 1 | mean | 1 |
| 387 | Lu12-15790233 | 12 | 15790233 | C/G | 0.83 | GLM | 1 | 2012 | 1 |
| 389 | Lu12-16036187 | 12 | 16036187 | A/G | 7.5 | GLM | 1 | mean | 1 |
| 389 | Lu12-16056974 | 12 | 16056974 | A/C | 7.4 | GLM | 1 | mean | 1 |
| 400 | Lu13-1950067 | 13 | 1950067 | A/G | 1.35 | GLM | 1 | mean | 1 |
| 400 | Lu13-1950274 | 13 | 1950274 | A/C | 2.9 | GLM | 1 | mean | 1 |
| 400 | Lu13-1950313 | 13 | 1950313 | G/A | 1.86 | GLM | 1 | mean | 1 |
| 403 | Lu13-2007694 | 13 | 2007694 | C/A | 3.22 | GLM | 1 | mean | 1 |
| 406 | Lu13-2008254 | 13 | 2008254 | G/A | 2.89 | GLM | 1 | mean | 1 |
| 406 | Lu13-2008290 | 13 | 2008290 | C/A | 3.77 | GLM | 1 | mean | 1 |
| 407 | Lu13-2012060 | 13 | 2012060 | T/G | 6.17 | GLM | 1 | mean | 1 |
| 409 | Lu13-2017210 | 13 | 2017210 | C/T | 3.45 | GLM | 1 | mean | 1 |
| 409 | Lu13-2017235 | 13 | 2017235 | C/G | 3.49 | GLM | 1 | mean | 1 |
| 409 | Lu13-2017392 | 13 | 2017392 | G/A | 2.88 | GLM | 1 | mean | 1 |
| 413 | Lu13-2461075 | 13 | 2461075 | C/A | 1.82 | GLM | 1 | mean | 1 |
| 413 | Lu13-2468744 | 13 | 2468744 | C/A | 1.62 | GLM | 1 | 2013 | 1 |
| 428 | Lu13-14295593 | 13 | 14295593 | G/C | 1.55 | GLM | 1 | mean | 1 |
| 428 | Lu13-14298794 | 13 | 14298794 | C/G | 2.16 | GLM | 1 | mean | 1 |
| 436 | Lu13-15568717 | 13 | 15568717 | T/A | 1.8 | GLM | 1 | mean | 1 |
| 450 | Lu14-1489925 | 14 | 1489925 | G/A | 0.27 | pLARmEB | 1 | 2015 | 1 |
| 478 | Lu14-18799890 | 14 | 18799890 | G/A | 2.89 | ISIS EM-BLASSO | 1 | 2013 | 1 |
| 481 | Lu15-992553 | 15 | 992553 | C/T | 3.14 | GLM | 1 | mean | 1 |
| 481 | Lu15-993525 | 15 | 993525 | G/A | 4.23 | GLM | 1 | mean | 1 |
| 483 | Lu15-2544402 | 15 | 2544402 | A/C | 2.22 | GLM | 1 | mean | 1 |
| 485 | Lu15-3661936 | 15 | 3661936 | G/A | 3.77 | GLM | 1 | mean | 1 |
