# Supplementary material for: Genome-Wide Association Studies for Pasmo Resistance in Flax (Linum usitatissimum L.)
Source: Front Plant Sci. 2019 Jan 14;9:1982. doi: 10.3389/fpls.2018.01982 (PMC6339956; doi:10.3389/fpls.2018.01982)
Supplement: Supplementary file 6 [file Table_6.docx]

**Supplementary table**

**Table S6** Number of QTL with positive-effect alleles (NPQTL) and pasmo severity of flax morphotypes.

| **Morphotype** | **No of accessions** | **NPQTL** | | | **Pasmo severity** | | |
| --- | --- | --- | --- | --- | --- | --- | --- |
|  |  | **Mean** | **Range** | ***s*** | **Mean** | **Range** | ***s*** |
| Fibre | 80 | 30.4 | 11-60 | 13.8 | 5.0 | 2-8 | 1.5 |
| Linseed | 290 | 14.5 | 3-43 | 4.7 | 6.6 | 1.8-9 | 1.0 |
| All | 370 | 18.0 | 3-60 | 10.0 | 6.2 | 1.8-9 | 1.3 |

s: standard deviation.
