# Supplementary material for: Genome-Wide Association Studies for Pasmo Resistance in Flax (Linum usitatissimum L.)
Source: Front Plant Sci. 2019 Jan 14;9:1982. doi: 10.3389/fpls.2018.01982 (PMC6339956; doi:10.3389/fpls.2018.01982)
Supplement: Supplementary file 7 [file Table_7.docx]

**Supplementary table**

**Table S7** Number of QTL with positive-effect alleles (NPQTL) in 370 accessions as related to morphotype and clustering of QTL and accessions.

| **QTL No** | **Tag QTN** | **QTL cluster** | **Total NPQTL (%)** | **NPQTL in Fibre (%)** | **NPQTL in Linseed** | **NPQTL in accession cluster 1** | **NPQTL in accession cluster 2** | **NPQTL in accession cluster 3** | **NPQTL in accession cluster 4** | **P value for χ2 test** | ***R*^2^** |
| --- | --- | --- | --- | --- | --- | --- | --- | --- | --- | --- | --- |
| 3 | Lu2-3803775 | 1 | 254(69) | 63(79) | 191 | 190 | 29 | 17 | 18 | 0.03905 | 3.32 |
| 14 | Lu4-17214936 | 1 | 306(83) | 70(88) | 236 | 222 | 33 | 25 | 26 | 0.2651 | 5.81 |
| 13 | Lu4-17204590 | 1 | 304(82) | 70(88) | 234 | 220 | 33 | 25 | 26 | 0.2136 | 5.17 |
| 63 | Lu13-13051094 | 1 | 285(77) | 76(95) | 209 | 187 | 37 | 35 | 26 | 3.094e-05 | 7.96 |
| 8 | Lu4-13306407 | 1 | 336(91) | 73(91) | 263 | 235 | 40 | 35 | 26 | 1 | 4.58 |
| 54 | Lu12-5552631 | 1 | 323(87) | 80(100) | 243 | 223 | 40 | 34 | 26 | 0.0002481 | 7.1 |
| 21 | Lu6-2081466 | 1 | 282(76) | 78(98) | 204 | 184 | 39 | 33 | 26 | 9.479e-07 | 8.3 |
| 10 | Lu4-14576826 | 1 | 205(55) | 54(68) | 151 | 131 | 35 | 18 | 21 | 0.01974 | 7.99 |
| 11 | Lu4-14615685 | 1 | 113(31) | 42(53) | 71 | 64 | 28 | 1 | 20 | 2.872e-06 | 10.85 |
| 64 | Lu13-14299019 | 1 | 189(51) | 57(71) | 132 | 138 | 27 | 0 | 24 | 7.817e-05 | 8.28 |
| 59 | Lu12-16358216 | 1 | 216(58) | 54(68) | 162 | 158 | 28 | 8 | 22 | 0.08161 | 4.25 |
| 51 | Lu12-474480 | 1 | 237(64) | 60(75) | 177 | 170 | 33 | 9 | 25 | 0.008217 | 8.33 |
| 55 | Lu12-5795458 | 1 | 234(63) | 75(94) | 159 | 169 | 39 | 0 | 26 | 3.813e-10 | 9.67 |
| 56 | Lu12-5819991 | 2 | 189(51) | 59(74) | 130 | 100 | 36 | 34 | 19 | 8.382e-06 | 6.9 |
| 61 | Lu13-2016767 | 2 | 136(37) | 36(45) | 100 | 62 | 23 | 33 | 18 | 0.1104 | 5.12 |
| 66 | Lu15-995626 | 2 | 128(35) | 32(40) | 96 | 71 | 5 | 28 | 24 | 0.31 | 6.27 |
| 12 | Lu4-14738243 | 2 | 134(36) | 45(56) | 89 | 47 | 32 | 35 | 20 | 4.508e-05 | 12.64 |
| 42 | Lu9-1430465 | 2 | 103(28) | 51(64) | 52 | 32 | 24 | 24 | 23 | 1.804e-15 | 10.76 |
| 53 | Lu12-2719326 | 2 | 113(31) | 52(65) | 61 | 32 | 25 | 34 | 22 | 1.156e-13 | 9.9 |
| 2 | Lu1-28707496 | 2 | 91(25) | 35(44) | 56 | 23 | 20 | 32 | 16 | 1.379e-05 | 5.7 |
| 28 | Lu7-2491132 | 3 | 112(30) | 41(51) | 71 | 72 | 19 | 0 | 21 | 7.604e-06 | 8.05 |
| 26 | Lu7-2452981 | 3 | 102(28) | 21(26) | 81 | 77 | 8 | 0 | 17 | 0.8756 | 6.3 |
| 27 | Lu7-2453965 | 3 | 101(27) | 29(36) | 72 | 70 | 18 | 0 | 13 | 0.05895 | 7.03 |
| 47 | Lu9-16348319 | 3 | 134(36) | 36(45) | 98 | 87 | 25 | 14 | 8 | 0.08634 | 4.64 |
| 49 | Lu10-8700793 | 3 | 157(42) | 59(74) | 98 | 96 | 33 | 3 | 25 | 3.52e-10 | 12.1 |
| 17 | Lu5-3575865 | 3 | 150(41) | 64(80) | 86 | 93 | 32 | 2 | 23 | 1.337e-15 | 9.64 |
| 18 | Lu5-4604607 | 3 | 97(26) | 36(45) | 61 | 59 | 15 | 2 | 21 | 3.029e-05 | 6.58 |
| 45 | Lu9-6270376 | 3 | 100(27) | 58(73) | 42 | 45 | 30 | 0 | 25 | 2.2e-16 | 14.34 |
| 1 | Lu1-9232234 | 3 | 92(25) | 54(68) | 38 | 48 | 21 | 1 | 22 | 2.2e-16 | 16.17 |
| 29 | Lu8-14317356 | 3 | 75(20) | 33(41) | 42 | 43 | 9 | 0 | 23 | 3.132e-07 | 14.32 |
| 62 | Lu13-11860250 | 3 | 56(15) | 27(34) | 29 | 30 | 7 | 0 | 19 | 3.951e-07 | 9.65 |
| 15 | Lu5-1554121 | 4 | 84(23) | 40(50) | 44 | 43 | 23 | 0 | 18 | 3.661e-10 | 7.75 |
| 67 | Lu15-8714776 | 4 | 78(21) | 43(54) | 35 | 30 | 23 | 0 | 25 | 2.072e-15 | 15.04 |
| 16 | Lu5-1650980 | 4 | 54(15) | 22(28) | 32 | 26 | 18 | 3 | 7 | 0.0004412 | 6.61 |
| 35 | Lu8-17270785 | 4 | 46(12) | 27(34) | 19 | 18 | 19 | 0 | 9 | 2.359e-10 | 9.59 |
| 6 | Lu3-22688547 | 4 | 60(16) | 24(30) | 36 | 35 | 11 | 0 | 14 | 0.0003101 | 8.98 |
| 30 | Lu8-15830073 | 4 | 66(18) | 24(30) | 42 | 40 | 6 | 2 | 18 | 0.002329 | 8.48 |
| 32 | Lu8-15841885 | 4 | 36(10) | 21(26) | 15 | 16 | 6 | 0 | 14 | 6.005e-08 | 8.35 |
| 31 | Lu8-15837449 | 4 | 33(9) | 18(23) | 15 | 15 | 3 | 0 | 15 | 4.379e-06 | 8.24 |
| 20 | Lu5-13500692 | 4 | 35(9) | 22(28) | 13 | 13 | 10 | 0 | 12 | 1.831e-09 | 11.9 |
| 24 | Lu6-15455712 | 4 | 28(8) | 18(23) | 10 | 9 | 4 | 0 | 15 | 4.62e-08 | 9.63 |
| 25 | Lu6-15506450 | 4 | 23(6) | 17(21) | 6 | 5 | 3 | 0 | 15 | 1.649e-09 | 12.62 |
| 23 | Lu6-14738507 | 4 | 20(5) | 17(21) | 3 | 1 | 5 | 0 | 14 | 1.047e-11 | 13.34 |
| 36 | Lu8-17749357 | 4 | 38(10) | 21(26) | 17 | 16 | 7 | 0 | 15 | 3.22e-07 | 10.16 |
| 37 | Lu8-18251174 | 4 | 29(8) | 21(26) | 8 | 6 | 9 | 0 | 14 | 2.29e-11 | 10.38 |
| 38 | Lu8-18447612 | 4 | 34(9) | 23(29) | 11 | 8 | 10 | 1 | 15 | 3.531e-11 | 11.66 |
| 34 | Lu8-16366918 | 4 | 33(9) | 21(26) | 12 | 13 | 5 | 0 | 15 | 3.185e-09 | 10.9 |
| 33 | Lu8-15963249 | 4 | 29(8) | 21(26) | 8 | 8 | 8 | 0 | 13 | 2.29e-11 | 14.22 |
| 48 | Lu9-19857367 | 4 | 26(7) | 17(21) | 9 | 8 | 6 | 0 | 12 | 7.67e-08 | 12.67 |
| 46 | Lu9-15527375 | 4 | 34(9) | 23(29) | 11 | 12 | 18 | 0 | 4 | 3.531e-11 | 6.76 |
| 22 | Lu6-5837358 | 4 | 38(10) | 21(26) | 17 | 14 | 10 | 0 | 14 | 3.22e-07 | 9.36 |
| 50 | Lu11-3330783 | 4 | 33(9) | 15(19) | 18 | 10 | 2 | 8 | 13 | 0.001101 | 7.09 |
| 60 | Lu13-1919638 | 4 | 33(9) | 28(35) | 5 | 6 | 12 | 0 | 15 | 2.2e-16 | 13.67 |
| 52 | Lu12-1621325 | 4 | 16(4) | 11(14) | 5 | 5 | 4 | 0 | 7 | 1.236e-05 | 9.41 |
| 40 | Lu8-23142500 | 4 | 32(9) | 19(24) | 13 | 9 | 5 | 0 | 18 | 1.958e-07 | 13.34 |
| 39 | Lu8-23104696 | 4 | 30(8) | 20(25) | 10 | 7 | 5 | 0 | 18 | 1.736e-09 | 16.53 |
| 41 | Lu9-1258326 | 4 | 35(9) | 27(34) | 8 | 8 | 5 | 0 | 22 | 3.09e-16 | 16.01 |
| 19 | Lu5-4858045 | 4 | 22(6) | 19(24) | 3 | 2 | 2 | 0 | 18 | 2.149e-13 | 12.83 |
| 5 | Lu3-20781286 | 4 | 26(7) | 22(28) | 4 | 1 | 8 | 0 | 17 | 4.326e-15 | 14.63 |
| 4 | Lu3-19643168 | 4 | 20(5) | 15(19) | 5 | 2 | 6 | 0 | 12 | 1.324e-08 | 12.82 |
| 7 | Lu4-37769 | 4 | 35(9) | 22(28) | 13 | 15 | 2 | 0 | 18 | 1.831e-09 | 11.23 |
| 58 | Lu12-16056974 | 4 | 40(11) | 27(34) | 13 | 13 | 8 | 0 | 19 | 3.868e-13 | 11.26 |
| 65 | Lu15-976617 | 4 | 34(9) | 26(33) | 8 | 12 | 2 | 0 | 20 | 2.121e-15 | 16.08 |
| 57 | Lu12-15686833 | 4 | 30(8) | 23(29) | 7 | 7 | 4 | 0 | 19 | 1.275e-13 | 13.9 |
| 44 | Lu9-4333365 | 4 | 28(8) | 27(34) | 1 | 2 | 4 | 0 | 22 | 2.2e-16 | 23.39 |
| 9 | Lu4-13779313 | 4 | 27(7) | 21(26) | 6 | 6 | 4 | 0 | 17 | 1.086e-12 | 13.72 |
| 43 | Lu9-1896658 | 4 | 27(7) | 26(33) | 1 | 1 | 6 | 0 | 20 | 2.2e-16 | 17.12 |

(a) The Pearson's Chi-squared test with Yates' continuity correction to test whether QTL effects are related to morphotypes. The order of QTL in the table is the same with that in **Figure 7**.
