# Supplementary material for: Genome-Wide Association Studies for Pasmo Resistance in Flax (Linum usitatissimum L.)
Source: Front Plant Sci. 2019 Jan 14;9:1982. doi: 10.3389/fpls.2018.01982 (PMC6339956; doi:10.3389/fpls.2018.01982)
Supplement: Supplementary file 8 [file Table_8.DOCX]

**Supplementary figure**

**Figure** **S1** The variances explained by the first 20 principal components (PCs) in the principal component analysis of 258,873 SNPs.

**
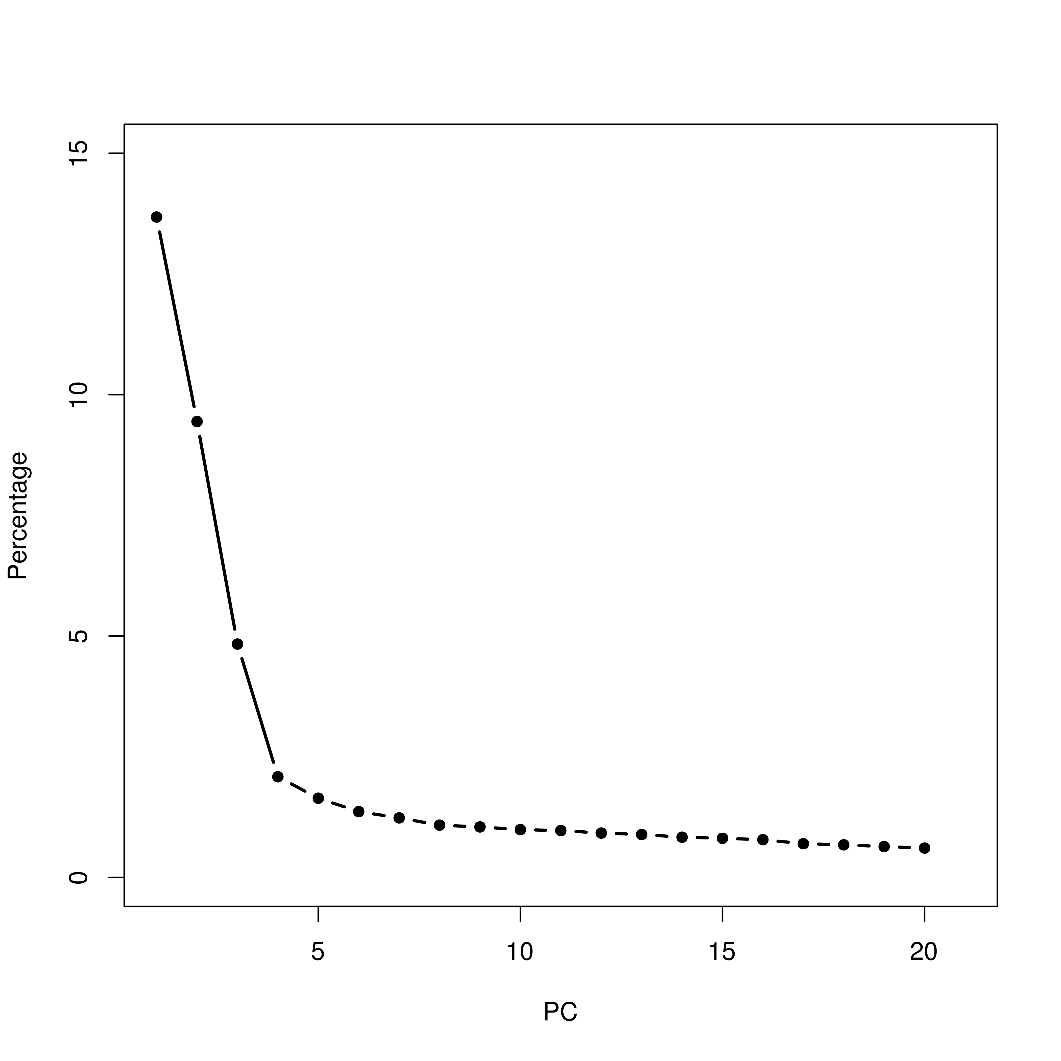
**
